# Piezo1 regulates meningeal lymphatic vessel drainage and alleviates excessive CSF accumulation

**DOI:** 10.1038/s41593-024-01604-8

**Published:** 2024-03-25

**Authors:** Dongwon Choi, Eunkyung Park, Joshua Choi, Renhao Lu, Jin Suh Yu, Chiyoon Kim, Luping Zhao, James Yu, Brandon Nakashima, Sunju Lee, Dhruv Singhal, Joshua P. Scallan, Bin Zhou, Chester J. Koh, Esak Lee, Young-Kwon Hong

**Affiliations:** 1 Department of Surgery, Norris Comprehensive Cancer Center, Keck School of Medicine, University of Southern California, Los Angeles, CA USA; 2 Department of Biochemistry and Molecular Medicine, Norris Comprehensive Cancer Center, Keck School of Medicine, University of Southern California, Los Angeles, CA USA; 3 Nancy E. and Peter C. Meinig School of Biomedical Engineering, Cornell University, Ithaca, NY USA; 4 Division of Plastic and Reconstructive Surgery, Beth Israel Deaconess Medical Center, Harvard Medical School, Boston, MA USA; 5 Department of Molecular Pharmacology and Physiology, University of South Florida, Tampa, FL USA; 6 New Cornerstone Science Laboratory, State Key Laboratory of Cell Biology, Shanghai Institute of Biochemistry and Cell Biology, Center for Excellence in Molecular Cell Science, Chinese Academy of Sciences, University of Chinese Academy of Sciences, Shanghai, China; 7 Division of Pediatric Urology, Texas Children’s Hospital, Baylor College of Medicine, Houston, TX USA

**Keywords:** Developmental disorders, Hydrocephalus, Cardiovascular biology, Brain

## Abstract

Piezo1 regulates multiple aspects of the vascular system by converting mechanical signals generated by fluid flow into biological processes. Here, we find that Piezo1 is necessary for the proper development and function of meningeal lymphatic vessels and that activating Piezo1 through transgenic overexpression or treatment with the chemical agonist Yoda1 is sufficient to increase cerebrospinal fluid (CSF) outflow by improving lymphatic absorption and transport. The abnormal accumulation of CSF, which often leads to hydrocephalus and ventriculomegaly, currently lacks effective treatments. We discovered that meningeal lymphatics in mouse models of Down syndrome were incompletely developed and abnormally formed. Selective overexpression of Piezo1 in lymphatics or systemic administration of Yoda1 in mice with hydrocephalus or Down syndrome resulted in a notable decrease in pathological CSF accumulation, ventricular enlargement and other associated disease symptoms. Together, our study highlights the importance of Piezo1-mediated lymphatic mechanotransduction in maintaining brain fluid drainage and identifies Piezo1 as a promising therapeutic target for treating excessive CSF accumulation and ventricular enlargement.

## Abstract (web summary)

The authors find that Piezo1 stimulation enhances meningeal lymphatics and boosts CSF drainage to treat hydrocephalus and ventriculomegaly, showing promise in Down syndrome and hydrocephalus models.

## Main

Lymphatic vessels are essential in tissue fluid homeostasis, immune cell trafficking and cellular waste clearance^1^. Lymphatic drainage refers to the process of absorbing fluid, cells, ions, large molecules and metabolites (collectively called lymph fluids) into lymphatic vessels, followed by their transport to the blood circulation after passing through a sequence of lymph nodes (LNs). The recent discovery and characterization of a new mechanically activated cation channel, Piezo1, provided a novel conceptual and experimental breakthrough toward a better understanding of how the lymphatics sense the mechanical stimulations generated by fluid flow. The Piezo1 channel senses the external physical forces applied to the cell membrane and cytoskeleton^2–7^. Mutations in Piezo1 have been linked to lymphatic disorders^8–10^. Recently, we reported that lymphatic mechanotransduction controlled by Piezo1 plays an essential role in lymphatic development, maintenance and function^11–14^. Piezo1 incorporates mechanical signals generated by laminar or oscillatory fluid flow into the genetic programs governing sprouting lymphangiogenesis^13^ and lymphatic valve formation^14^, respectively. Yoda1 is a small molecule agonist for Piezo1 (ref. ^15^) and enhances the channel’s sensitivity to mechanical forces by lowering its mechanical threshold for activation^16^. Our studies revealed that Yoda1 can trigger lymphatic mechanotransduction through Piezo1 and mimic the flow-related phenotypes in vitro and in animals^13,14^.

The central nervous system has long been considered devoid of a lymphatic drainage system^17^. However, structured lymphatic networks in the meningeal layer have been recently reported^18,19^. These meningeal lymphatic vessels (mLVs) were found to be crucial for maintaining brain health by regulating the cerebral fluid balance, facilitating immune responses and removing cellular and metabolic wastes^20–24^. mLVs transport cerebrospinal fluid (CSF) and immune cells to the regional LNs, including the cervical and mandibular LNs^18,19^. Notably, structural degeneration and functional decline of mLVs in aging leads to mLV dysfunction^25^, which has been associated with the progression of age-related neurological disorders, such as Alzheimer’s disease and Parkinson’s disease^22,26–29^. In addition, brain fluid drainage through mLVs is also critical for immune surveillance and injury recovery in the central nervous system^30–34^. Accordingly, these studies have established a robust conceptual groundwork, suggesting that improving the functionality of mLVs could be a potential therapeutic strategy against these diseases.

Hydrocephalus is a disease with enlarged ventricles and an abnormal accumulation of CSF^35,36^. Structural abnormalities, infection, injury, tumors, bleeding or other medical conditions are the reported causes^37^. Enlarged ventricles are often associated with elevated intracranial pressure (ICP), but the pressure can often be within the normal range as in normal-pressure hydrocephalus (NPH), especially in older individuals^38^. Treatments for hydrocephalus include invasive neurosurgeries that create a new drainage route for the fluid, but these dramatic procedures can result in postsurgical complications^39^. Ventriculomegaly is another neurological disease characterized by ventricular enlargement with excessive CSF accumulation^40^. Multiple reasons, including brain shrinkage, anatomic obstruction, CSF overproduction or infection, may contribute to the initiation and progression of the disease. Notably, ventriculomegaly has also been associated with mice and humans with chromosomal defects, such as Down syndrome (DS), to varying degrees and incidences^41–45^; it may present mild clinical symptoms with normal ICP but can progress to hydrocephalus.

This report presents a compelling set of data demonstrating the crucial role of Piezo-controlled lymphatic mechanotransduction in normal brain fluid drainage. Our study revealed that Piezo1 is essential for the development and function of mLVs. Whereas lymphatic knockout of *Piezo1* led to mLV malformation, impaired CSF drainage and ventricular enlargement with elevated ICP, genetic and chemical activation of Piezo1 enhanced brain fluid drainage. We further extended our findings toward suppressing the pathological phenotypes of mouse models of hydrocephalus and ventriculomegaly by targeting Piezo1 in mLVs.

## Results

### Piezo1 is essential for mLV development

**Fig. 1:**
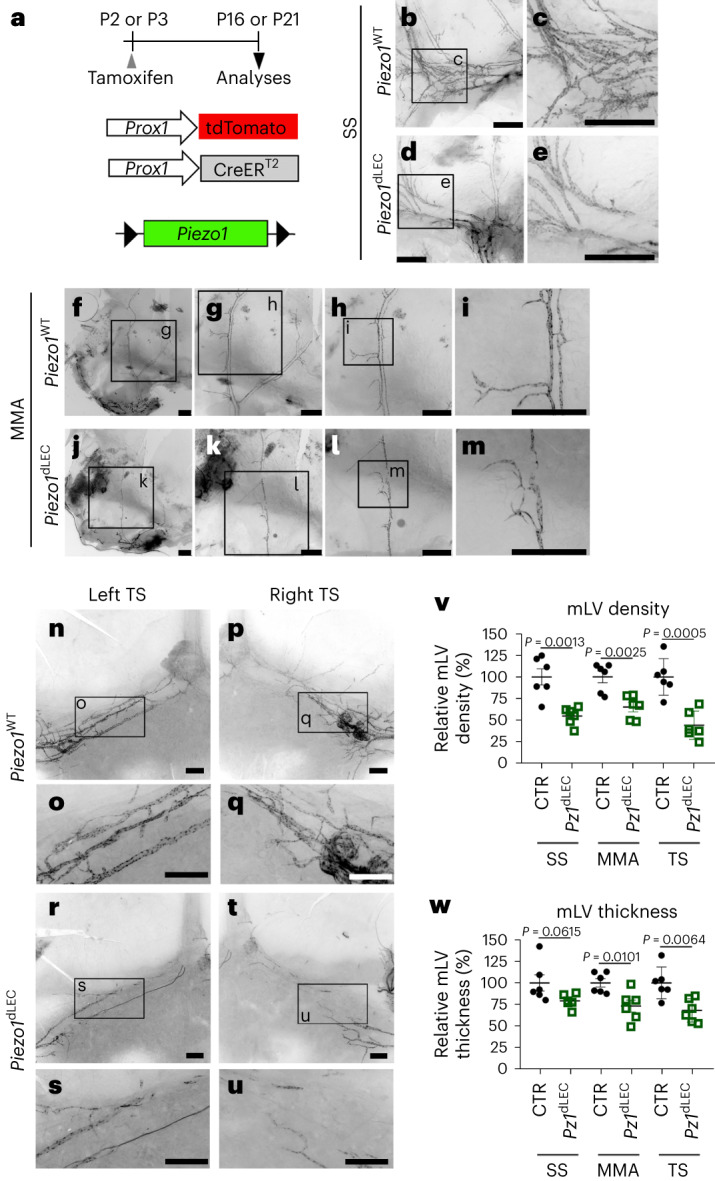
Piezo1 is required for mLV development. **a**, Diagram depicting the experimental strategy for conditional lymphatic *Piezo1* deletion. Tamoxifen (50 mg per kg (body weight)) was subcutaneously injected into neonatal pups at postnatal day 2 (P2) or P3. Wild-type *Piezo1* (*Piezo1*^WT^) control (CTR) mice have *Prox1-*tdTomato, *Prox1*-CreER^T2^ and *Piezo1*^+/+^ alleles, whereas *Piezo1*^dLEC^ mice have *Prox1-*tdTomato, *Prox1*-CreER^T2^ and *Piezo1*^fl/fl^ alleles. **b**–**u**, Mice were killed at P16 (**b**–**m**) or P21 (**n**–**u**), and their meninges were collected to capture mLV images. mLVs were visualized by the red fluorescent signals produced by the *Prox1-*tdTomato lymphatic reporter allele. **v**,**w**, Relative vessel density (**v**) and thickness (**w**) of mLVs in the sigmoid sinus (SS; **b**–**e**), middle meningeal artery (MMA) region (**f**–**m**) and transverse sinus (TS) region (**n**–**u**) in control or *Piezo1*^dLEC^ mice are shown; scale bars, 500 µm; *Pz1*^dLEC^, *Piezo1*^dLEC^ mice. Each data point represents one mouse; *n* = 6 mice per group. Data were analyzed by two-tailed *t*-test and are presented as mean values ± s.e.m. Source data

**Extended Data Fig. 1:**
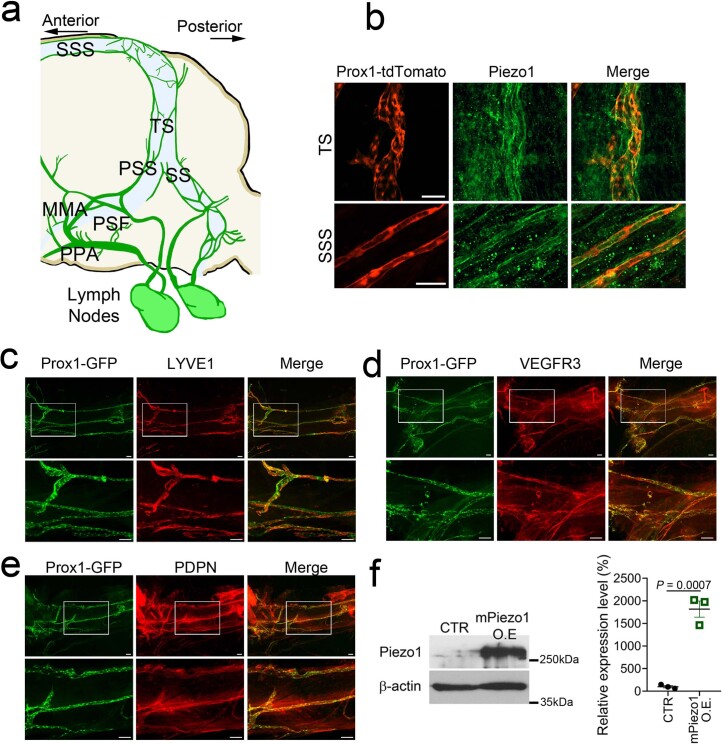
Expression of Piezo1 in Mouse Meningeal Lymphatic Vessels. (**a**) Diagram illustrating the distribution of mouse meningeal lymphatic vessels (mLVs). SSS, Superior Sagittal Sinus; TS, Transverse Sinus; PSS, Petrosquamosal Sinus; SS, Sigmoid Sinus; PSF, Petrosquamous Fissure; MMA, Middle Meningeal Artery; PPA, Pterygopalatine Artery. (**b**) Expression of Piezo1 protein in mLVs. The meninges were isolated from adult *Prox1*-tdTomato mice and subjected to whole-mount staining with a rabbit anti-Piezo1 polyclonal antibody (generated by the authors). Scale bars, 50 µm. (**c-e**) Expression of LYVE1, VEGFR3, and PDPN in mLVs. The meninges from *Prox1*-EGFP mice were whole-mount stained with anti-LYVE1, anti-VEGFR3, or anti-PDPN antibodies. Scale bars, 100 µm. (**f**) Specificity test of the rabbit anti-Piezo1 polyclonal antibody. HEK293 cells were transiently transfected with a control vector or a mouse Piezo1-expressing vector. After 48 hours, western blot analyses were performed against the cell lysates using the rabbit anti-Piezo1 polyclonal antibody or a rabbit anti-beta actin antibody. Statistics: two-tailed t-test. Source data

We have previously reported the crucial role of Piezo1 in regulating the mechanotransduction pathway involved in lymphatic development and function^13,14^. Here, we sought to extend the previous conceptual framework and evaluate the impact of Piezo1-regulated lymphatic mechanotransduction on brain fluid homeostasis. Toward this aim, we first confirmed the expression of Piezo1 and other lymphatic markers in mLVs of *Prox1*-tdTomato lymphatic reporter mice^46^ (Extended Data Fig. 1). We next induced conditional deletion of *Piezo1* in lymphatic endothelial cells (LECs) of neonatal mice with *Prox1*-CreER^T2^, *Piezo1*^fl/fl^ and *Prox1*-tdTomato alleles, denoted *Piezo1*^dLEC^ mice (Fig. 1a), and found that lymphatic *Piezo1* deletion disrupted mLV development, which was characterized by reduced vessel branches, disorganized and hypomorphic vascular networks and fragmented trunks (Fig. 1b–w). We verified the efficient deletion of *Piezo1* expression in the lymphatics of *Piezo1*^dLEC^ mice following intraperitoneal (i.p.) tamoxifen administration by assessing the loss of both mRNA and protein expression of Piezo1 in mLVs (Supplementary Figs. 1 and 2). Moreover, we confirmed that tamoxifen treatment alone did not alter mLV development and drainage function in *Prox1*-CreER^T2^ mice (Supplementary Fig. 3). Together, these data indicate that Piezo1 is required for proper development of mLVs in mice.

### Lymphatic *Piezo1* knockout impairs brain fluid drainage and causes ventricular enlargement

**Fig. 2:**
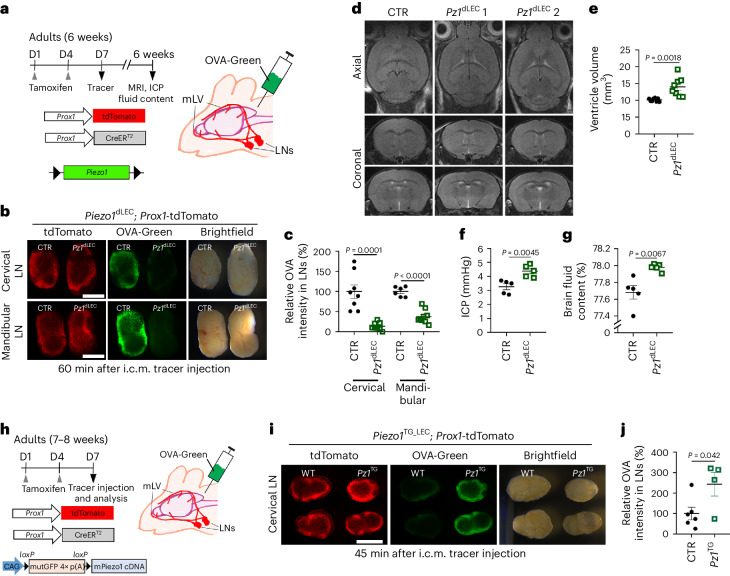
Effects of altered lymphatic Piezo1 expression on brain fluid drainage and CSF homeostasis. **a**, Experimental scheme. Adult wild-type control (*Prox1*-CreER^T2^; *Piezo1*^+/+^; *Prox1*-tdTomato) and lymphatic *Piezo1* deletion mice (*Prox1-*CreER^T2^; *Piezo1*^fl/fl^; *Prox1*-tdTomato) were i.p. injected with tamoxifen (50 mg per kg (body weight) twice, 3 days apart) at the age of 6 weeks. Three days after the second tamoxifen administration, a brain fluid tracer (ovalbumin Alexa Fluor 488 conjugate (OVA-Green); 3.5 µl, 0.5 mg ml^–1^) was i.c.m. injected. After 60 min, the cervical and mandibular LNs were collected and imaged; D, day. **b**, Fluorescence images of the collected cervical and mandibular LNs; scale bar, 0.5 mm. **c**, Quantification of the relative intensity of OVA-Green signal in the LNs. One data point represents the sum of the fluorescence intensity of the left and right LNs of a single mouse; *n* = 6–9 mice per group. **d**–**g**, Control and lymphatic *Piezo1*-mutant pups (*Prox1*-CreER^T2^; *Piezo1*^fl/fl^) were subcutaneously injected with tamoxifen (50 mg per kg (body weight)) at P4. After 6 weeks, brain MRI was performed, magnetic resonance images were acquired (**d**), and ventricular volumes (**e**) were measured. Three-dimensional rendered images of the brain MRI are shown (Extended Data Fig. 3). ICP (**f**) and relative brain fluid content (**g**) were assessed 1 day after MRI. In **e**–**g**, each data point corresponds to a single mouse; *n* = 5–8 mice per group. **h**–**j**, Targeted lymphatic Piezo1 overexpression promotes brain fluid tracer clearance. **h**, Experimental scheme showing adult wild-type control (*Prox1*-CreER^T2^; *Prox1*-tdTomato) and lymphatic *Piezo1*-transgenic (*Piezo1*^TG_LEC^) mice (*Prox1*-CreER^T2^; *Piezo1*^TG^; *Prox1*-tdTomato) injected i.p. with tamoxifen (50 mg per kg (body weight) twice, 3 days apart) at the age of 7–8 weeks. Three days later, a green fluorescent tracer (OVA-Green) was i.c.m. injected. After 45 min, the cervical LNs were collected and imaged. The *Piezo1* transgenic overexpression construct contains a nonfunctional mutant eGFP gene and four tandem copies of poly(A) (p(A)) sequences flanked by *loxP* sequences^13^. **i**, Fluorescence images of the collected cervical LNs; scale bar, 1 mm. **j**, Relative intensity of OVA-Green signal in the LNs was quantified. Each data point represents the sum of the fluorescence intensity of LNs on the right and left sides of a single mouse; *n* = 4–6 mice per group. *Pz1*^dLEC^, *Piezo1*^dLEC^ mice; *Pz1*^TG^, *Piezo1*^TG_LEC^ mice. Data were analyzed by two-tailed *t*-test and are presented as mean values ± s.e.m. Source data

**Extended Data Fig. 2:**
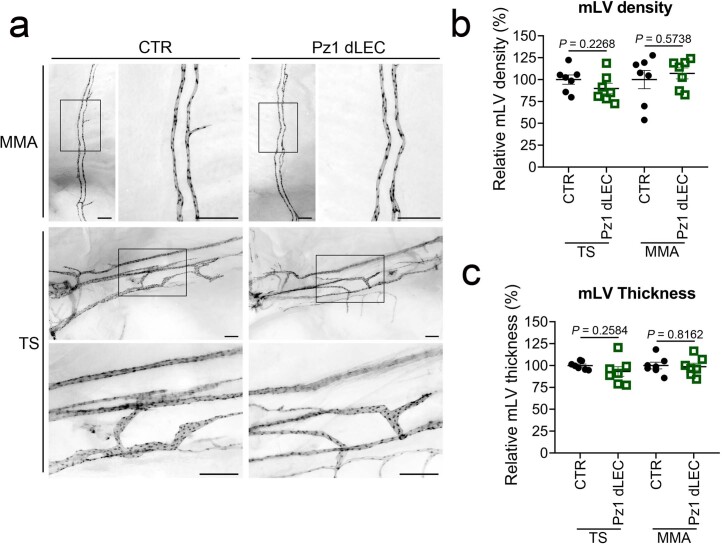
Short-term Deletion of *Piezo1* Doesn’t Lead to Significant Morphological Changes in Adult Meningeal Lymphatics. Adult wild-type control (CTR, *Prox1*-tdTomato) mice and lymphatic *Piezo1* null mice (*Pz1* dLEC) (*Piezo1*^dLEC^, *Prox1*-tdTomato) were i.p. injected with Tamoxifen (50 mg/kg, twice, three days apart) at the age of 8 weeks. (**a**) mLV images were captured six days after the first Tamoxifen injection. Scale bars: 200 µm. (**b**, **c**) Lymphatic vascular density (**b**) and thickness (**c**) in the control and *Pz1* dLEC mice (n = 7 mice /group) were quantitated and compared. Statistics: two-tailed *t*-test. Data are presented as mean values +/- SEM. Source data

**Extended Data Fig. 3:**
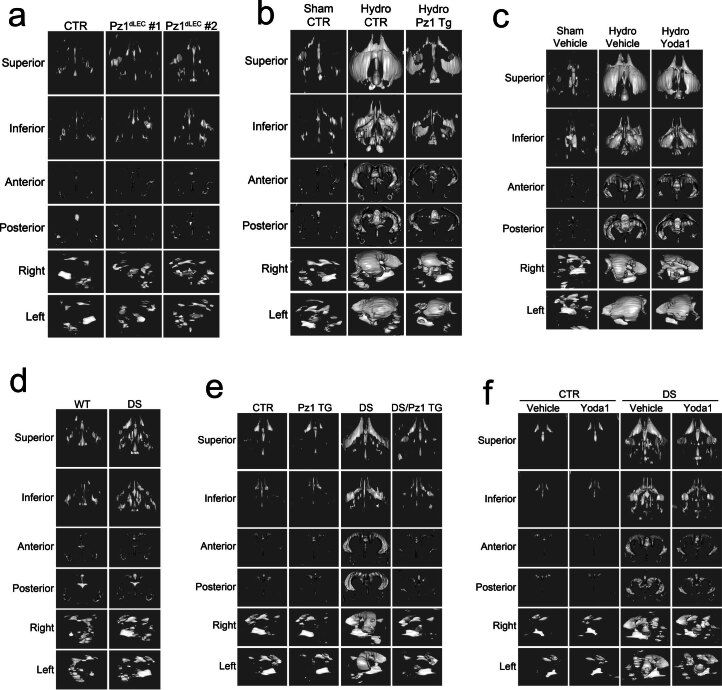
3-D Rendering of MRI Scans for Ventricular Volume Measurements. The two-dimensional MRI brain images presented in Fig. 2d, Fig. 5e, Fig. 6b, Fig. 7b, m, and Fig. 8e of the main figure section were rendered to three-dimensional representations in panels (**a**) through (**f)** using the Multi-image Analysis GUI (Mango) to display the volume of the ventricles. See Methods for details.

**Extended Data Fig. 4:**
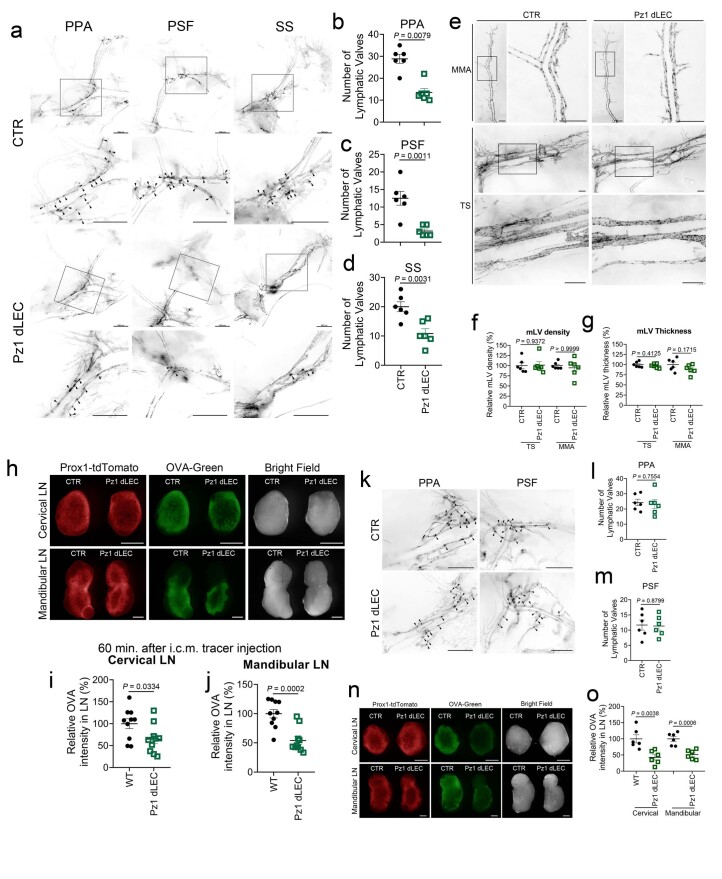
Essential Roles of the mLV Intraluminal Valves in Brain Fluid Drainage. (**a-j**) *Targeting Lymphatic Valves during the Developmental Phase in Neonates*. Control mice (CTR) (*Prox1*-CreER^T2^; *Piezo1*^+/+^; *Prox1*-tdTomato) and lymphatic *Piezo1* KO mice (*Pz1* dLEC) (*Prox1*-CreER^T2^; *Piezo1*^flox/flox^; *Prox1*-tdTomato) were subcutaneously injected with Tamoxifen at a low dose (15 mg/kg) at P1 to target deletion of *Piezo1* in lymphatic valves. The mice were euthanized at P21 for further analyses of lymphatic valves and vessels. (**a**) Microscopic images showing the presence of lymphatic valves (arrowheads) in the basal mLVs around Pterygopalatine Artery (PPA), Petrosquamous Fissure (PSF), and Sigmoid Sinus (SS). Scale bars: 500 µm. (**b-d**) Number of lymphatic valves in these areas was counted. n = 6 mice/group. (**e**) Images showing the absence of morphological changes of mLVs in mice (P21) treated with low dose Tamoxifen. Scale bars: 200 µm. The lymphatic vascular density (**f**) and thickness (**g**) in the control and *Pz1* dLEC mice showed no significant differences. n = 6 mice/group (**h**) A brain fluid tracer (OVA-Green: Ovalbumin, Alexa Fluor™ 488 Conjugate) was i.c.m. injected at P21. The cervical and mandibular LNs were harvested and imaged 60 minutes after the tracer injection. Note dramatically diminished tracer drainages. Scale bars: 500 µm. (**I**, **j**) The relative OVA intensity in LNs was quantified. n = 10 mice/group. (**k-o**) *Targeting Matured Lymphatic Valves in Adult Mice*: Control (CTR) and *Pz1* dLEC mice (6 months old) were i.p. injected with Tamoxifen at a low dose (15 mg/kg, twice, three days apart). After 6 days from the first tamoxifen administration, a brain fluid tracer (OVA-Green: Ovalbumin, Alexa Fluor™ 488 Conjugate) was i.c.m. injected, and the mice were euthanized to analyze lymphatic valves and brain fluid drainage. Lymphatic valves (arrowheads) around Pterygopalatine Artery (PPA) and Petrosquamous Fissure (PSF) were imaged (**k**) and their numbers were counted (**I**, **m**). n = 6 mice/group. (**n**) After 60 minutes from the tracer injection, the cervical and mandibular LNs were harvested and imaged. Scale bars: 500 µm. (**o**) The relative intensity of OVA-Green in LNs was quantified. n = 6 mice/group. Each data point represents the sum of the fluorescence intensity of LNs on the right and left sides of a single mouse (**h, n**). Statistics: (**b, f**) two-tailed Mann-Whitney U test. (**c, d, g, i, j, l, m, o**) two-tailed *t*-test. Statistics: two-tailed *t*-test. Data are presented as mean values +/- SEM. Source data

We next examined the function of Piezo1 in brain fluid drainage through mature mLVs in adult mice (6 weeks old). We first induced lymphatic *Piezo1* deletion by i.p. tamoxifen administration to control and *Piezo1*^dLEC^ mice on days 1 and 4 and assessed its effects on brain fluid drainage to the CSF-draining LNs on day 7. For this, we performed intracisterna magna (i.c.m.) injections of a green fluorescent tracer and determined the amount of the tracer drained to the cervical and mandibular LNs 60 min after i.c.m. injection (Fig. 2a). We noted a marked decrease in the amount of tracer drained to the LNs when *Piezo1* was deleted in lymphatics (Fig. 2b,c), indicating the essential role of lymphatic Piezo1 in brain fluid drainage. Of note, *Piezo1* deletion for this short period (6 days) in adult mice did not induce any detectable changes in the morphology of lymphatic vessels or values (Extended Data Fig. 2), suggesting that the diminished drainage observed after short-term *Piezo1* deletion is likely due to immediate functional impairments in lymphatic vessels rather than progressive structural degenerations that were observed in our previous study^13^. We next examined the longer-term effects of lymphatic *Piezo1* deletion on brain fluid drainage in young-adult *Piezo1*^dLEC^ mice and found that 6-week-long lymphatic *Piezo1* deletion led to a substantial expansion in ventricular volume, elevated ICP and increased brain fluid accumulation (Fig. 2d–g and Extended Data Fig. 3). Although basal mLVs are equipped with lymphatic valves^25^, their contribution to brain fluid drainage remains unclear. Thus, we next investigated the role of mLV valves in brain fluid drainage by targeting *Piezo1* of lymphatic valves. We have previously reported that low-dose tamoxifen treatment of *Piezo1*^dLEC^ neonatal mice caused preferential malformation of lymphatic valves without significant morphological alterations on mesentery lymphatics^14^. Consistently, *Piezo1*^dLEC^ neonates, treated with low-dose tamoxifen, revealed profoundly reduced numbers of lymphatic valves in basal mLVs near the pterygopalatine artery, petrosquamous fissure and sigmoid sinus and did not show any significant changes in the density and thickness of mLVs (Extended Data Fig. 4a–g). Indeed, we noted markedly diminished brain tracer drainage when mLV valves were not properly formed, highlighting the essential role of lymphatic valves in the drainage function of basal mLVs (Extended Data Fig. 4h–j). Similarly, we administered low-dose tamoxifen to adult *Piezo1*^dLEC^ mice, which had fully matured lymphatic valves within their mLVs. After 6 days, we assessed the integrity of these valves and examined their effects on brain fluid drainage. Notably, whereas the fully developed lymphatic valves in adult mice did not show any noticeable morphological changes, there was still a detectable reduction in brain fluid drainage (Extended Data Fig. 4k–o), suggesting that *Piezo1* deletion in the mature lymphatic valves of mLVs may rapidly reduce brain drainage efficiency even when their structural degeneration was not evident. Together, these findings demonstrate that Piezo1-controlled mechanotransduction is crucial for the structural formation of mLVs and their direct functions in regulating brain fluid drainage.

### Lymphatic overexpression of Piezo1 enhances brain fluid outflow

**Extended Data Fig. 5:**
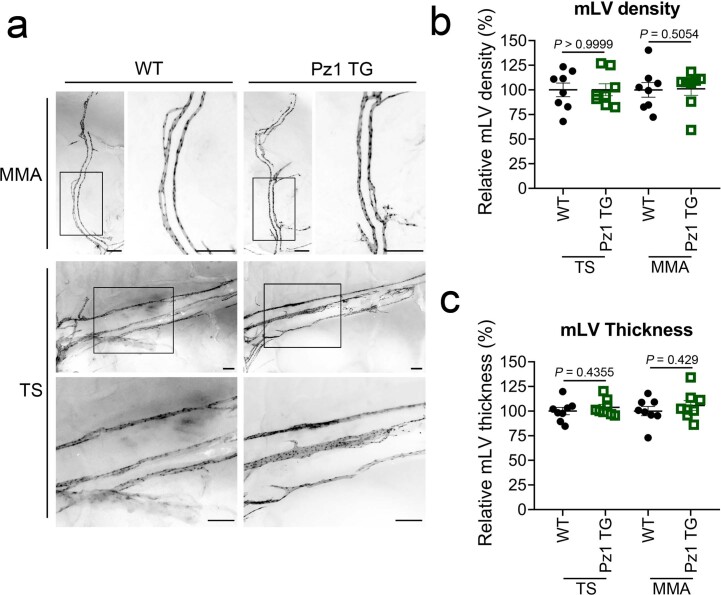
Absence of Detectable Lymphangiogenesis in Piezo1 Lymphatic Transgenic Mice After Brief Piezo1 Overexpression. Adult control (*Prox1*-CreER^T2^; *Prox1*-tdTomato) and lymphatic *Piezo1* transgenic mice (*Pz1* TG) (*Prox1*-CreER^T2^; *Piezo1*^TG^; *Prox1*-tdTomato) were i.p. injected with Tamoxifen (50 mg/kg, twice, three days apart) at the age of 10 weeks. mLV images were captured 6 days after the first Tamoxifen injection (**a**). Relative lymphatic vascular density (**b**) and thickness (**c**) in the control and *Pz1* TG mice (n = 8 mice/group) were quantitated. Scale bars: 200 µm. Statistics: (**b**) two-tailed Mann-Whitney U test. (**c**) two-tailed *t*-test. Data are presented as mean values +/- SEM. Source data

We next examined the impact of activating Piezo1 in lymphatics on CSF drainage. We ectopically overexpressed Piezo1 in LECs of adult *Piezo1*-transgenic mice (*Piezo1*^TG_LEC^)^13^ by tamoxifen administration on days 1 and 4 and conducted brain tracer drainage assays on day 7 (Fig. 2h). Piezo1 overexpression in mLVs of *Piezo1*^TG_LEC^ mice was verified by detecting the elevated levels of both Piezo1 mRNA and protein (Supplementary Figs. 1 and 2). Indeed, lymphatic Piezo1 overexpression resulted in a marked increase in tracer drainage to the cervical LNs, as visualized 45 min after i.c.m. injection of the tracer (Fig. 2i,j). Importantly, following Piezo1 overexpression over a short period (6 days), this drainage improvement occurred before detectable lymphatic expansion (Extended Data Fig. 5), implying that the drainage enhancement is likely due to immediate functional enhancement rather than progressive lymphatic growth.

### Systemic activation of Piezo1 effectively promotes brain fluid drainage

**Fig. 3:**
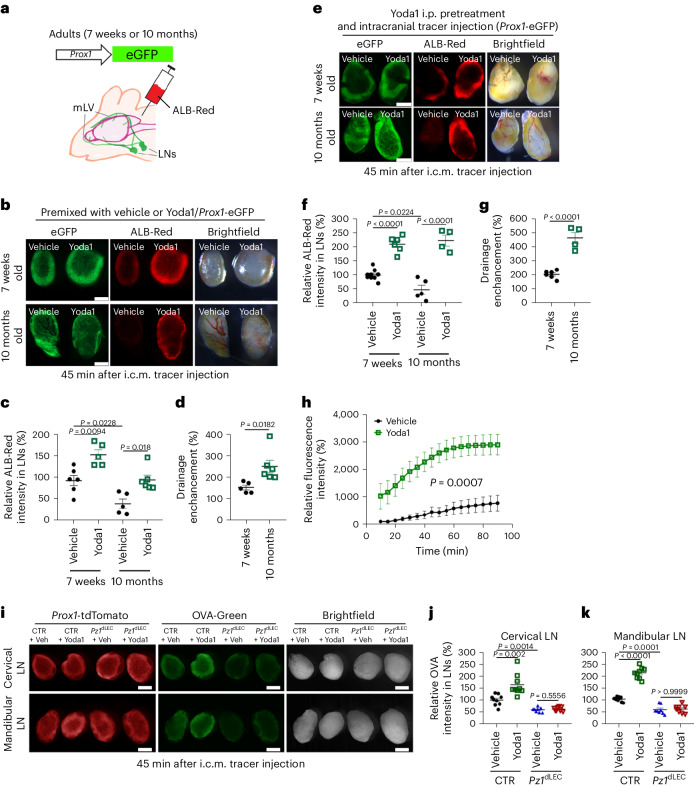
Local and systemic activation of Piezo1 stimulates brain fluid drainage. **a**–**c**, Experimental scheme (**a**). A red fluorescent tracer (albumin-Alexa Fluor 594 (ALB-Red)) was premixed with vehicle or Yoda1 (50 µM, final) and i.c.m. injected into *Prox1*-eGFP mice in two age groups (7 weeks old and 10 months old, five to six mice per group). After 45 min, the cervical LNs were collected and imaged (**b**), and their relative fluorescence intensities were graphed (**c**); *n* = 5–6 mice per group. Data were analyzed by one-way analysis of variance (ANOVA; *P* < 0.0001) followed by a Bonferroni multiple comparison test. **d**, Percent increase in drainage by Yoda1 treatment in each age group, which was calculated by dividing the intensity of individual Yoda1-treated LNs by the average intensity value of all vehicle-treated LNs; *n* = 5–6 mice per group. Data were analyzed by two-tailed *t*-test. **e**–**g**, Systemic Yoda1 pretreatment by i.p. injection expedited brain tracer drainage. *Prox1*-eGFP mice (7 weeks old and 10 months old) were i.p. injected with vehicle or Yoda1 (213 µg per kg (body weight)) at 18 h and 90 min before i.c.m. injection of ALB-Red. After 45 min, the cervical LNs were collected and imaged (**e**), and the relative intensity of drained tracer is shown (**f**). Each data point represents the sum of the fluorescence intensity of LNs on both sides of one mouse; *n* = 4–9 mice per group. Data were analyzed by one-way ANOVA (*P* < 0.0001) followed by a Bonferroni multiple comparison test. **g**, Drainage enhancement by Yoda1 in each age group; *n* = 4–9 mice per group. Data were analyzed by two-tailed *t*-test; scale bars, 500 µm (**b** and **e**). **h**, Time-lapse image analysis showing Yoda1-induced enhancement of brain tracer drainage. Mice were systemically pretreated (i.p. injection) with vehicle or Yoda1 (213 µg per kg (body weight)) at 18 h and 90 min before i.c.m. injection of Indocyanine Green (ICG). Time-lapse images of ICG drained to LNs were captured at the indicated times after injection (Extended Data Fig. 6), and the relative fluorescence intensity was graphed; *n* = 6 mice per group. Data were analyzed by two-way repeated measures ANOVA (*P* = 0.0007). **i**–**k**, Lymphatic *Piezo1* deletion abolishes the Yoda1-induced enhancement of CSF outflow. Adult control (*Prox1*-tdTomato) and lymphatic *Piezo1*-null (*Piezo1*^dLEC^; *Prox1*-tdTomato) mice were i.p. injected with tamoxifen (50 mg per kg (body weight) twice, 3 days apart) at the age of 8 weeks. A fluorescent tracer (OVA-Green), premixed with vehicle (Veh) or Yoda1 (50 µM, final), was i.c.m. injected into control and *Piezo1*^dLEC^ mice 6 days after the first injection of tamoxifen. After 45 min, LNs were collected and imaged (**i**). **j**,**k**, Quantification of relative tracer intensity in LNs (*n* = 8–9 mice). Each data point represents the total fluorescence intensity of LNs on both sides of one mouse; scale bars, 500 µm; *Pz1*^dLEC^, *Piezo1*^dLEC^ mice. Data were analyzed by Kruskal–Wallis *H*-test (*P* < 0.0001) followed by a Bonferroni multiple comparison test (statistical significance, *P* < 0.0083; **j**) and one-way ANOVA (*P* < 0.0001) followed by a Bonferroni multiple comparison test (**k**). Data are presented as mean values ± s.e.m. Source data

**Extended Data Fig. 6:**
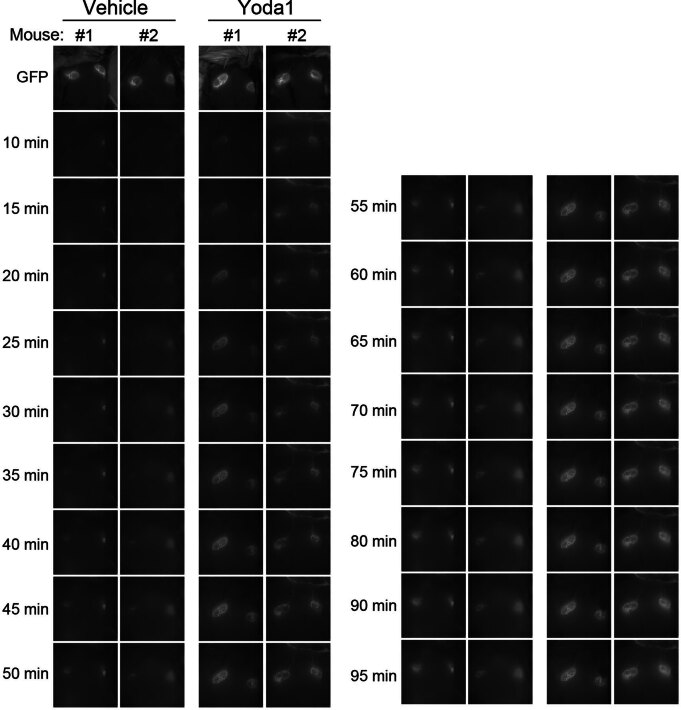
Systemic Yoda1 Administration Enhances Brain Fluid Tracer Outflow to Lymph Nodes. Time-lapse images showing lymphatic drainage of i.c.m. injected Indocyanine Green (ICG) to the mandibular LNs. Adult *Prox1-*EGFP mice (6-7 months, n = 6 mice/group) were i.p. injected with the vehicle or Yoda1 (213 µg/kg) at 18 hours and 90 minutes before ICG tracer injection (3.5 µL, 2.5 mg/mL). Time-lapse images of ICG tracer accumulated to the LNs were captured from 10 to 95 minutes post ICG injection. The GFP signal serves as the locational reference of the left- and right-side mandibular LNs. The relative amount of drained ICG was quantified and charted in Fig. 3h in the main figure section.

**Extended Data Fig. 7:**
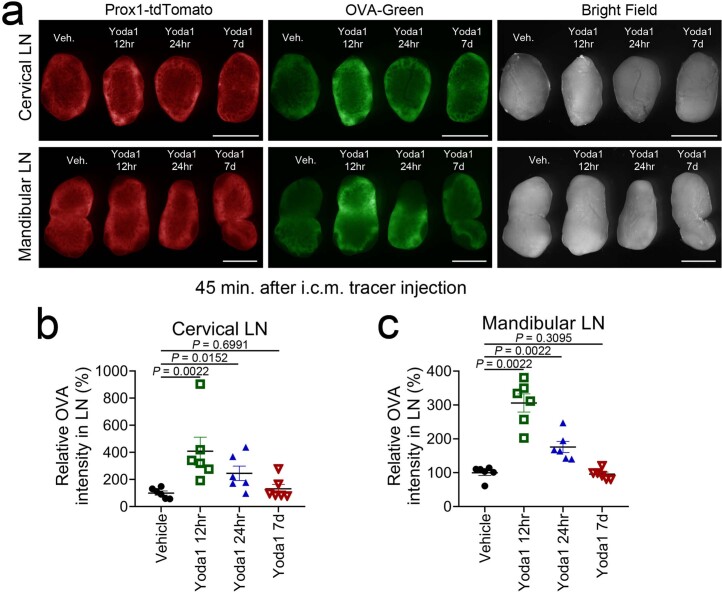
The Yoda1 Effect on Brain Fluid Outflow Persists Up to 24 Hours. Adult wild-type lymphatic reporter mice (*Prox1*-tdTomato, 9-10 months old) were i.p. injected with the vehicle or Yoda1 (213 µg/kg) at seven days, 24 hours, 12 hours before tracer i.c.m. injection (OVA-Green: Ovalbumin, Alexa Fluor™ 488 Conjugate). After 45 minutes post tracer injection, LNs were collected and imaged to visualize the drained tracer (**a**). (**b**, **c**) The relative tracer intensity in the cervical LNs (**b**) and mandibular LNs (**c**) was quantified (n = 6 mice/group). Each data point represents the sum of the fluorescence intensity of LNs on the right and left sides of a single mouse. Scale bars: 500 µm. Statistics: Kruskal-Wallis H test, p = 0.0032 (**b**); p = 0.0002 (**c**), followed by Bonferroni correction method (statistical significance, p < 0.0083). Data are presented as mean values +/- SEM. Source data

In addition to the targeted genetic activation of Piezo1 described above, we next set out to assess the effect of chemical activation of Piezo1 on brain fluid drainage. We i.c.m. injected a mixture of a fluorescent tracer and vehicle or Yoda1, a small compound agonist for Piezo1 (ref. ^15^), into *Prox1*-enhanced green fluorescent protein (*Prox1*-eGFP) reporter mice^47^ and compared tracer drainage efficiency (Fig. 3a). Indeed, when mixed with Yoda1, a greater quantity of tracer was drained to the cervical LNs in 45 min than when mixed with vehicle (Fig. 3b,c). This effect was more pronounced in 10-month-old mice than in 7-week-old mice because of the age-related decrease in brain fluid drainage (Fig. 3d), which was also reported previously^25,28^. We then investigated the effect of systemic Yoda1 administration on brain tracer drainage. *Prox1*-eGFP reporter mice received i.p. injections of vehicle or Yoda1 at 18 h and 90 min before the brain tracer assay. Indeed, systemic Yoda1 pretreatment markedly enhanced brain tracer outflow, and this effect was again more evident in 10-month-old mice than in 7-week-old mice due to age-associated decline of mLV function (Fig. 3e–g). In addition, in vivo time-lapse imaging revealed that, compared to vehicle treatment, systemic Yoda1 pretreatment remarkably enhanced brain tracer outflow (Fig. 3h and Extended Data Fig. 6). This Yoda1 effect of enhancing brain tracer drainage was found to persist for at least 24 h (Extended Data Fig. 7). Importantly, we confirmed that the Yoda1-induced enhancement of CSF outflow requires the presence of Piezo1 expression in lymphatics. Lymphatic *Piezo1* deletion was induced in adult control and *Piezo1*^dLEC^ mice by tamoxifen administration, and, after 6 days, brain fluid drainage assays were performed using a fluorescent tracer dye that was premixed with vehicle or Yoda1. Indeed, lymphatic *Piezo1* deletion in *Piezo1*^dLEC^ mice abrogated the Yoda1-mediated enhancement of brain fluid drainage (Fig. 3i–k), suggesting that the Yoda1 effect on brain fluid outflow is mediated mainly through Piezo1 in mLVs. Together, these data demonstrate that systemic Yoda1 treatment effectively promotes brain fluid outflow through the activation of Piezo1 expressed in mLVs of mice.

### Mechanistic understanding of the Piezo1-mediated activation of fluid drainage function

**Fig. 4:**
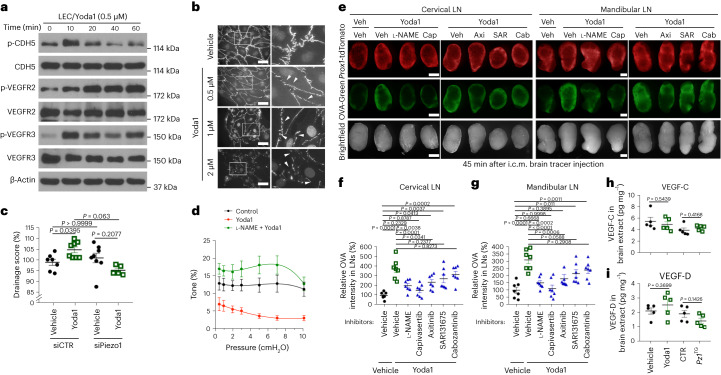
Enhanced lymphatic drainage and transport by Piezo1 activation. **a**, Increased phosphorylation of CDH5 (Try 658), VEGFR2 (Tyr 1054/Tyr 1059) and VEGFR3 (Tyr 1230/Tyr 1231) in cultured primary human LECs in vitro treated with Yoda1 (0.5 µM, final). **b**, CDH5 immunofluorescence stains showing increased junctional gaps (arrowheads) in 2D LEC in vitro cultures after Yoda1 treatment at 0.5, 1 or 2 µM for 8 h; scale bars, 20 µm. **c**, Piezo1-dependent increase in drainage efficiency of engineered lymphatics in vitro. Primary LECs were transfected with control (scrambled) or Piezo1 short interfering RNA (siRNA) for 24 h, used to build lymphatics in 3D PDMS chips^50^ and treated with vehicle or Yoda1 (1 µM, final) to evaluate drainage capability. Detailed images for the engineered lymphatics are shown in Extended Data Fig. 9; siCTR and vehicle, *n* = 7 independent experiments; siCTR and Yoda1, *n* = 8 independent experiments; siPiezo1 and vehicle, *n* = 8 independent experiments; siPiezo1 and Yoda1, *n* = 5 independent experiments. Data were analyzed by one-way ANOVA (*P* = 0.0097) followed by a Bonferroni multiple comparison test. **d**, Lymphatic vessel contractility test. Surgically excised axillary collecting lymphatics were treated with vehicle, Yoda1 or l-NAME + Yoda1 at the indicated intraluminal pressures, and the percent vessel tone was measured. Additional functionality values are shown in Extended Data Fig. 10; *n* = 6 independent samples. Data were analyzed by two-way repeated measures ANOVA (*P* = 0.003) between treatments followed by Tukey’s multiple comparison test; *P* < 0.0001 between control and Yoda1; *P* = 0.0004 between control and l-NAME + Yoda1; *P* < 0.0001 between Yoda1 and l-NAME + Yoda1. **e**, Inhibition of Piezo1 downstream effectors suppresses Yoda1-induced promotion of brain tracer drainage. Fluorescent tracer (OVA-Green) was premixed with vehicle, Yoda1 (50 µM, final) or Yoda1 (50 µM, final) + inhibitors, such as l-NAME (eNOS), capivasertib (Cap; AKT), axitinib (Axi; VEGFR1–VEGFR3), SAR131675 (SAR; VEGFR3) or cabozantinib malate (Cab; VEGFR2; 258 µg ml^–1^ final concentration of each inhibitor), and i.c.m. injected into *Prox1*-tdTomato mice (15 weeks old). After 45 min, LNs were collected and imaged; scale bars, 500 µm. **f**,**g**, Quantification of relative tracer intensity in cervical (**f**) and mandibular (**g**) LNs (*n* = 7 mice per group). One data point represents the sum of the fluorescence intensity of LNs of both sides of a single mouse. Data were analyzed by one-way ANOVA (*P* < 0.0001) followed by Tukey’s multiple comparison test. **h**,**i**, Protein expression levels of VEGF-C (**h**) and VEGF-D (**i**) were measured in the whole brains of mice that were treated with vehicle or Yoda1 (213 µg per kg (body weight) two times at 18 h and 90 min before tissue collection) or in the whole brains of control or *Piezo1*^TG_LEC^ mice that were i.p. injected with tamoxifen (50 mg per kg (body weight) twice, 3 days apart) at the age of 8 weeks. *Pz1*^TG^, *Piezo1*^TG_LEC^ mice. Each data point represents one mouse (*n* = 5 mice per group). Data were analyzed by two-tailed *t*-test and are presented as mean values ± s.e.m. Source data

**Extended Data Fig. 8:**
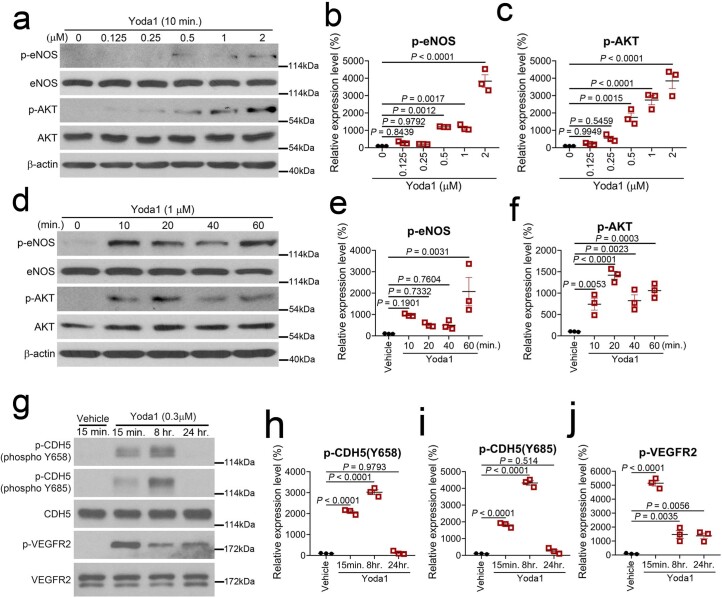
AKT1, eNOS, CDH5, and VEGFR2 Proteins Are Rapidly Phosphorylated in Cultured Lymphatic Endothelial Cells Upon Yoda1 Treatment. (**a-f**) Phosphorylation of eNOS (Ser1177) and AKT1 (Ser473) in cultured human primary dermal LECs after Yoda1 treatment at different concentrations for 10 minutes (**a**) or at a fixed concentration (1 µM) for different periods (**d**). The ratio of phosphorylated protein over the entire protein is expressed (**b, c, e, f**). (**g-j**) Phosphorylation of CDH5 (Y658, Y685) and VEGFR2 (Tyr1054/1059) in cultured human LECs after Yoda1 (0.3 µM) treatment for the indicated periods (**g**). The phosphorylation level relative to the entire protein is quantified (**h-j**). Experiments were repeated three times. Statistics: one-way ANOVA, p < 0.0001, followed by Dunnett’s multiple comparison test. Data are presented as mean values +/- SEM. Source data

**Extended Data Fig. 9:**
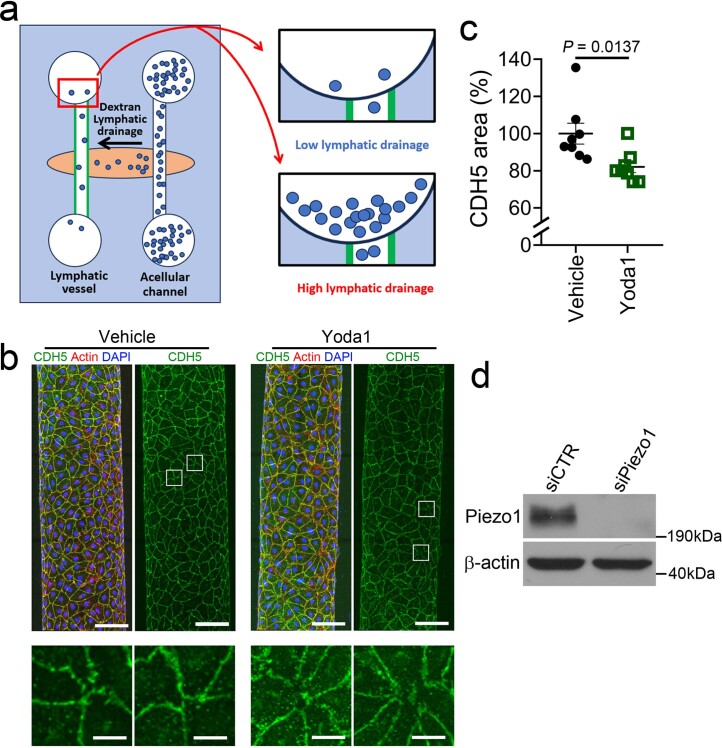
Piezo1 Activation Decreases the CDH5-stained Area and Increases Drainage of Lymphatic Vessel Mimetics in Polydimethylsiloxane (PDMS) Chip. **(a)** Schematic illustration of the 3-D lymphatic vessel model used for this study. The outcome of the Yoda1-induced drainage increase is presented in Fig. 4c. **(b)** Fluorescence confocal images of the engineered lymphatic vessels stained for F-actin and CDH5. Enlarged CDH5 images (boxed) show more discontinuous junctions in the Yoda1-treated group than in the vehicle group. Scale bars: 100 μm (10 μm, enlarged images). Acellular channels are not shown. **(c)** The relative area of CDH5-stained cellular junctions (n = 8 independent experiments). Statistics: two-tailed t-test. **(d)** Western blot assays verifying the efficient knock-down of Piezo1 in LECs prepared for the drainage measurement shown in Fig. 4c (n = 4 independent samples). Data are presented as mean values +/- SEM. Source data

**Extended Data Fig. 10:**
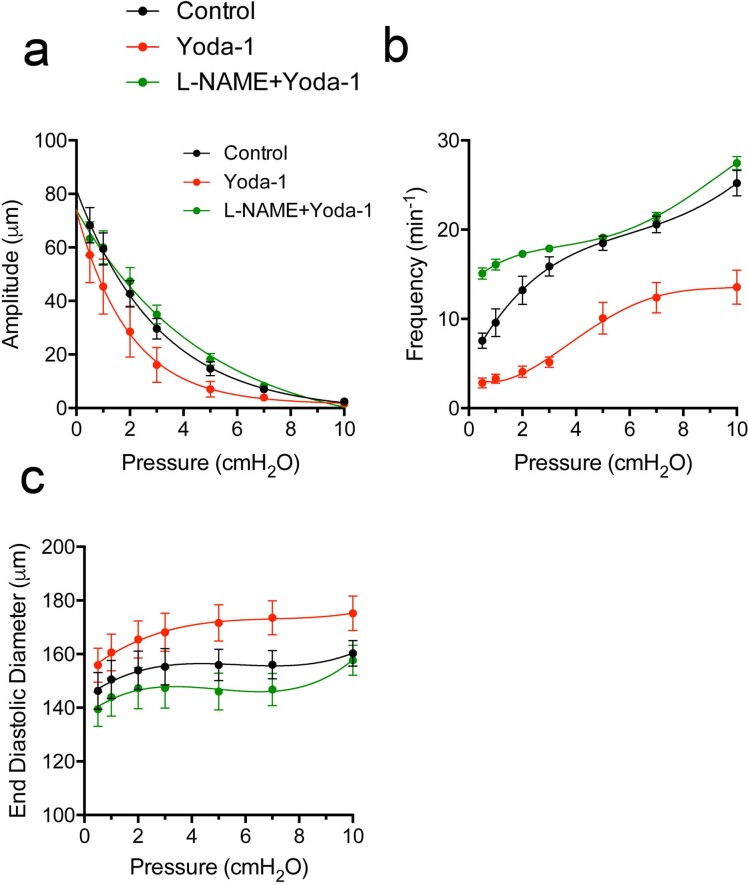
Yoda1 Modifies the Contractile Characteristics of Collecting Lymphatics via an eNOS-Dependent Mechanism. Axillary collecting lymphatic vessel was surgically excised and exposed to the vehicle, Yoda1 (1 µM), or L-NAME (100 µM) +Yoda1 (1 µM). Lymphatic vessel contractile amplitude (**a**), frequency (**b**), and end-diastolic diameter (**c**) were measured while intraluminal pressure was stepped from 0.5 cmH_2_O to 10 cmH_2_O as indicated. The enclosed videos display the changes in the contractile parameters of an excised collecting lymphatic when exposed to the vehicle, Yoda1, and L-NAME+Yoda1 (See Supplementary Videos S1-S6). n = 6 independent samples. Statistics: two-way repeated measure ANOVA, p = 0.2217 (**a**); p < 0.0001 (**b**); p = 0.1145 (**c**) between treatments. Tukey’s multiple comparison test for frequency measures followed, p < 0.0001 between control vs. Yoda1; p = 0.0138 between control vs. L-NAME+Yoda1; p < 0.0001 between Yoda1 vs. L-NAME+Yoda1 (**b**). Data are presented as mean values +/- SEM. Source data

We next investigated the molecular and cellular mechanisms underlying enhanced lymphatic drainage triggered by treatment with Yoda1. When treated with submicromolar levels of Yoda1, cultured primary human LECs rapidly induced the phosphorylation of CDH5, vascular endothelial growth factor receptor 2 (VEGFR2), VEGFR3, AKT1 and endothelial nitric oxide synthase (eNOS) in vitro (Fig. 4a, Supplementary Fig. 4a–c and Extended Data Fig. 8). Notably, the expression levels of Prox1 and Lyve1 were not altered in LECs by Yoda1 treatment in vitro (Supplementary Fig. 5a–c). At the cellular level, Yoda1 promoted the formation of discontinuous junctional gaps, a hallmark of increased lymphatic drainage^48,49^, in two-dimensional (2D) LEC cultures, and this phenotype was not caused by potential chemical cytotoxicity of Yoda1 at these concentrations (Fig. 4b and Supplementary Figs. 4d,e and 5d). The Yoda1-induced LEC junctional change was also detectable in three-dimensional (3D) initial lymphatics grown in polydimethylsiloxane (PDMS) chips^50^ (Extended Data Fig. 9a–c). Consistent with the increased junctional discontinuity, Yoda1-treated engineered lymphatics showed an enhanced in vitro tracer drainage function in a Piezo1-dependent manner (Fig. 4c and Extended Data Fig. 9d). At the level of collecting lymphatics, Yoda1 treatment swiftly triggered the dilation of surgically extracted mouse collecting lymphatic vessels and decreased the frequency of their contractions. These two functional characteristics, which are recognized to support lymphatic transport^51^, were found to be dependent on eNOS activity that was enhanced by Yoda1 treatment (Fig. 4d, Extended Data Fig. 10 and Supplementary Videos 1–6). We next performed in vivo inhibition assays to evaluate the role of the downstream molecular players in Yoda1-triggered activation of brain fluid drainage. Inhibition of Piezo1 downstream effectors using various chemical inhibitors, such as *N*^G^-nitro-l-arginine methyl ester hydrochloride (l-NAME^52^; eNOS), capivasertib^53^ (AKT), axitinib^54^ (VEGFR1–VEGFR3), SAR131675 (ref. ^55^; VEGFR3) and cabozantinib^56^ (VEGFR2), in mice efficiently suppressed Yoda1-induced promotion of brain tracer drainage to LNs (Fig. 4e–g). Despite this, we did not find any significant increase in protein levels of VEGF-C and VEGF-D within the brain parenchyma of Yoda1-treated mice or *Piezo1*^TG_LEC^ mice (Fig. 4h,i), excluding the brain parenchyma as the major source of VEGFR ligands. Together, these results suggest that activating lymphatic Piezo1 enhances CSF outflow by increasing not only fluid absorption at the initial lymphatics but also fluid transport at the collecting lymphatics level, providing a mechanistic understanding of how Piezo1-controlled mechanotransduction enhances brain fluid drainage.

### Lymphatic Piezo1 overexpression suppresses hydrocephalus disease phenotypes in mice

**Fig. 5:**
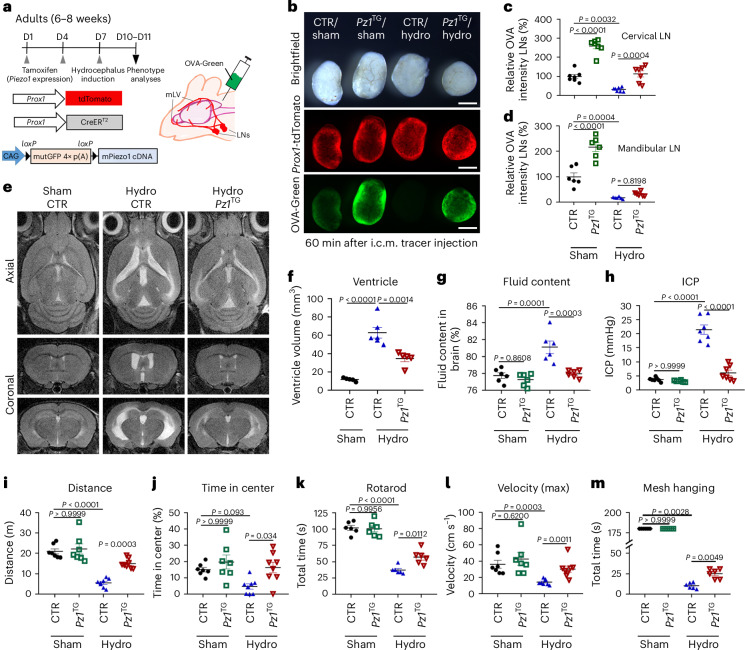
Lymphatic Piezo1 overexpression ameliorates hydrocephalus phenotypes. **a**, Adult wild-type control (*Prox1*-CreER^T2^; *Prox1*-tdTomato) or lymphatic *Piezo1*^TG_LEC^ (*Prox1*-CreER^T2^; *Prox1*-tdTomato; *Piezo1*^TG^) mice (6–8 weeks old) were i.p. injected with tamoxifen (50 mg per kg (body weight)) on days 1 and 4 to induce Piezo1 overexpression in lymphatics. On day 7, the kaolin-based hydrocephalus (hydro) model was established, as described in the Methods. **b**–**d**, On day 10, a fluorescent tracer (OVA-Green) was i.c.m. injected, and, after 60 min, the cervical and mandibular LNs were collected and imaged (**b**). The relative amounts of tracer drained to the cervical (**c**) and mandibular (**d**) LNs were quantified. One data point represents the sum of the fluorescence intensity of LNs of both sides of one mouse (*n* = 6–8 mice per group). Data were analyzed by one-way ANOVA (*P* < 0.0001) followed by Tukey’s multiple comparison test; scale bars, 500 µm. **e**,**f**, T2-weighted brain magnetic resonance images were captured on day 11 (**e**), and the ventricular volume was quantified (*n* = 5–6 mice per group; **f**). Three-dimensional rendered images of the brain MRI are shown (Extended Data Fig. 3). Data were analyzed by one-way ANOVA (*P* < 0.0001) followed by a Bonferroni multiple comparison test. **g**–**m**, Brain fluid content (**g**) and ICP (**h**) were measured on day 11, and physical activity (**i**–**m**) was evaluated on day 10 (*n* = 6–8 mice per group). Data were analyzed by one-way ANOVA (*P* < 0.0001) followed by Tukey’s multiple comparison test (**g** and **k**), one-way ANOVA (*P* < 0.0001 (**h** and **i**), *P* = 0.005 (**j**) and *P* = 0.002 (**l**)) followed by a Bonferroni multiple comparison test or Kruskal–Wallis *H*-test (*P* = 0.0006 (**l**) and *P* < 0.0001 (**m**)) followed by a Bonferroni multiple comparison test (statistical significance, *P* < 0.0083). Each data point represents one mouse, whereas one data point in **b** and **c** is the sum of the fluorescence intensity of the left and right LNs of one mouse. *Pz1*^TG^, *Piezo1*^TG_LEC^ mice. Data are presented as mean values ± s.e.m. Source data

We next explored the potential therapeutic efficacy of activating lymphatic Piezo1 to decrease abnormal CSF accumulation in hydrocephalus. We chose the kaolin-based hydrocephalus model due to its widespread use and detailed analysis in previous studies^57^. We first overexpressed Piezo1 in lymphatics of adult *Piezo1*-transgenic mice (*Piezo1*^TG_LEC^)^13^ by tamoxifen injection on days 1 and 4 and induced the kaolin-based hydrocephalus model on day 7. On days 10–11, we assessed the effect of lymphatic Piezo1 overexpression on hydrocephalus phenotypes, such as CSF physiology, ventricular volume and physical activity, in mice (Fig. 5a). Notably, we found that this hydrocephalus model was accompanied by impaired brain fluid outflow, enlarged ventricular volume, increased brain fluid content, elevated ICP and reduced physical activity (Fig. 5b–m and Extended Data Fig. 3). However, lymphatic Piezo1 overexpression before disease induction effectively alleviated these hydrocephalus-associated symptoms. Together, our study demonstrates that the brief preemptive overexpression of Piezo1 in lymphatics markedly reduces the severity of symptoms associated with hydrocephalus in a mouse model.

### Systemic Piezo1 activation reduces hydrocephalus disease phenotypes in mice

**Fig. 6:**
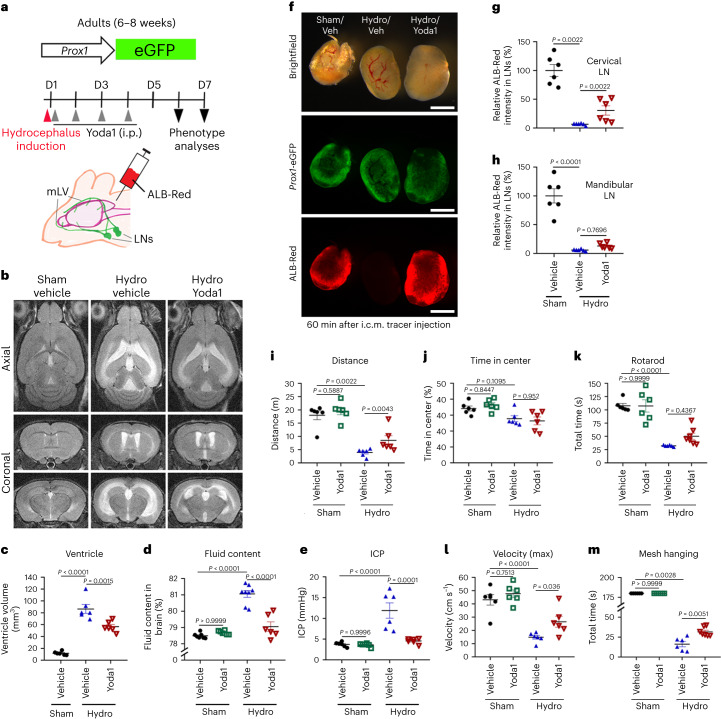
Yoda1-mediated activation of Piezo1 suppresses hydrocephalus phenotypes. **a**, Experimental scheme. The hydrocephalus model was induced in adult *Prox1*-eGFP mice (6–8 weeks old) on day 1, and mice were subsequently i.p. injected with Yoda1 (213 µg per kg (body weight) daily for 4 consecutive days from day 1). **b**,**c**, Brain magnetic resonance images were captured on day 6 (**b**), and ventricular volumes were calculated (**c**). Three-dimensional rendered images of the brain MRI are also shown in Extended Data Fig. 3. **d**,**e**, Brain fluid content (**d**) and ICP (**e**) were measured on day 6; *n* = 6–8 mice per group. Data were analyzed by one-way ANOVA (*P* < 0.0001) followed by Tukey’s multiple comparison test (**c** and **e**) or a one-way ANOVA (*P* < 0.0001) followed by a Bonferroni multiple comparison test (**d**). **f**–**h**, On day 7, a fluorescent tracer (ALB-Red) was i.c.m. injected, and, after 60 min, the cervical and mandibular LNs were imaged (**f**) and quantified for the amount of drained tracer (**g** and **h**, respectively); *n* = 6 mice per group. Data were analyzed by Kruskal–Wallis *H*-test (*P* < 0.0001) followed by a Bonferroni multiple comparison test (statistical significance, *P* < 0.0167; **g**) or one-way ANOVA (*P* < 0.0001) followed by a Tukey’s multiple comparison test (**h**); scale bars, 500 µm. **i**–**m**, Mice were subjected to physical activity tests on day 5. Each data point represents one mouse, whereas one data point in **g** and **h** represents the sum of the fluorescence intensity of the left and right LNs of one mouse; *n* = 6–7 mice per group. Data were analyzed by Kruskal–Wallis *H*-test (*P* = 0.0004 (**i**) and *P* < 0.0001 (**m**)) followed by a Bonferroni multiple correction test (statistical significance, *P* < 0.0083), one-way ANOVA (*P* = 0.0028 (**j**) and *P* < 0.0001 (**l**)) followed by a Tukey’s multiple testing correction or one-way ANOVA (*P* < 0.0001) followed by a Bonferroni multiple testing correction (**k**). Data are presented as mean values ± s.e.m. Source data

In a subsequent intervention study, we evaluated the therapeutic impact of Yoda1 on hydrocephalus symptoms. We first induced hydrocephalus in adult *Prox1*-eGFP reporter mice on day 1 and immediately i.p. treated them with vehicle or Yoda1 daily for four consecutive days (Fig. 6a). On days 6 and 7, mice underwent various phenotype evaluations, including brain magnetic resonance imaging (MRI), fluid content assessment and ICP measurements. Indeed, systemic Piezo1 activation by Yoda1 i.p. injection clearly reduced the extent of ventricular enlargement, CSF accumulation and ICP increase (Fig. 6b–e and Extended Data Fig. 3). Moreover, Yoda1 treatment restored impaired CSF outflow to the cervical and mandibular LNs and suppressed the loss of physical activities in the hydrocephalus model (Fig. 6f–m). These findings demonstrate that systemic Piezo1 activation through Yoda1 provides promising interventional effects toward acute hydrocephalus in mice.

### Brain lymphatic malformation and ventricular enlargement in a mouse model of DS

**Fig. 7:**
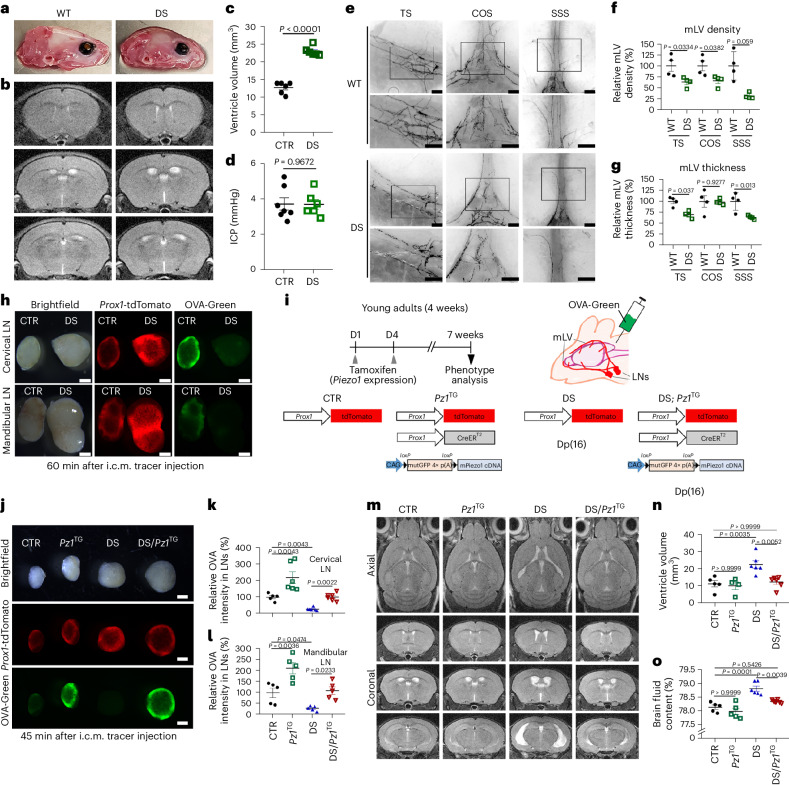
Lymphatic Piezo1 overexpression reduces meningeal lymphatic malformation and ventriculomegaly in mice with DS. **a**,**b**, Brightfield images showing distinct skull shapes (**a**) and T2-weighted brain MRIs displaying ventricular enlargement in Dp(16) mice with DS (**b**). Three-dimensional rendered images of the brain MRI are also shown in Extended Data Fig. 3. **c**,**d**, Ventricular volume (**c**) and ICP (**d**) of wild-type control and Dp(16) mice with DS. Each data point represents one mouse; *n* = 6–7 mice (6–8 weeks of age) per group. Data were analyzed by two-tailed *t*-test. **e**, mLVs in the transverse sinus (TS), confluence of sinuses (COS) and superior sagittal sinus (SSS) of wild-type (WT; *Prox1*-tdTomato) and Dp(16) mice with DS (Dp(16)1Yey; *Prox1*-tdTomato) were imaged (4 weeks old). **f**,**g**, Vessel density (**f**) and thickness (**g**) of mLVs were compared between wild-type mice and mice with DS (*n* = 4 mice per group). Data were analyzed by two-tailed *t*-test. Each data point in all graphs represents one mouse. **h**, A green fluorescent tracer (OVA-Green) was i.c.m. injected into control mice (*Prox1*-tdTomato) and mice with DS (Dp(16)1Yey; *Prox1*-tdTomato; 4 weeks old). After 60 min, the cervical and mandibular LNs were collected and imaged (*n* = 4 mice per group); scale bars, 500 µm. **i**–**o**, Lymphatic Piezo1 overexpression suppresses ventricular enlargement in a DS model. **i**, Experimental scheme. Young-adult control (*Prox1*-tdTomato), *Piezo1*^TG_LEC^ (*Prox1*-tdTomato; *Prox1*-CreER^T2^; *Piezo1*^TG^), DS (*Prox1*-tdTomato; Dp(16)1Yey/+) and DS/*Piezo1*^TG_LEC^ (Dp(16)1Yey/+; *Prox1*-tdTomato; *Prox1*-CreER^T2^; *Piezo1*^TG^; 4 weeks old) mice were i.p. injected with tamoxifen (day 1 and day 4) to induce lymphatic Piezo1 overexpression. **j**, After 7 weeks, the OVA-Green tracer was i.c.m. injected, and the cervical and mandibular LNs were collected and imaged after 45 min; scale bars, 500 µm. Longer-exposure images revealed a distinct difference in tracer drainage amounts between control mice and mice with DS (Supplementary Fig. 6a). **k**,**l**, Relative OVA-Green intensity was quantified for the cervical (**k**) and mandibular (**l**) LNs. One data point represents the sum of the fluorescence intensity of the left and right LNs of one mouse; *n* = 5–6 mice per group. Data were analyzed by Kruskal–Wallis *H*-test (*P* = 0.0003) followed by a Bonferroni multiple testing correction (statistical significance, *P* < 0.0083; **k**) or a one-way ANOVA (*P* < 0.0001) followed by a Tukey’s multiple comparison test (**l**). **m**–**o**, Additional sets of mice were equally prepared and subjected to brain MRI (**m**), ventricular volume assessment (**n**) and brain fluid content quantification (**o**). Three-dimensional rendered images of the brain MRI are also shown in Extended Data Fig. 3; scale bars, 500 µm; *n* = 4–6 mice per group. Data were analyzed by one-way ANOVA (*P* = 0.0007 (**n**) and *P* < 0.0001 (**o**)) followed by a Bonferroni multiple testing correction. Each data point represents one mouse, whereas one data point in **c** and **d** represents the sum of the fluorescence intensity of the left and right LNs of one mouse. Data are presented as mean values ± s.e.m. *Pz1*^TG^, *Piezo1*^TG_LEC^ mice. Source data

We next used a genetic model of chronic brain fluid accumulation to reinforce our findings from the surgery (kaolin)-based acute model of pathological CSF buildup in mice. Previous studies have indicated different degrees of ventricular enlargement and excessive CSF accumulation in infants and adults with DS^41–44^. Similarly, mouse models of DS have been reported to exhibit enlarged ventricles from an early age^58–60^. Consistent with these reports, we also noted that mice with DS (Dp(16)1Yey/+)^59^ displayed ventricular enlargement with the normal range of ICP (Fig. 7a–d and Extended Data Fig. 3). In addition, multiple previous studies have reported disturbed lymphatic development in mouse embryos and human fetuses with DS^61–64^. Indeed, our study uncovered that mLVs in Dp(16) mice with DS were markedly underdeveloped or malformed and were characterized by reduced density and thickness of mLVs (Fig. 7e–g). In line with these morphological abnormalities, mLVs in Dp(16) mice with DS also exhibited substantially diminished brain tracer drainage to the cervical and mandibular LNs (Fig. 7h). Together, these findings indicate that disturbed development of mLVs may be a key factor leading to reduced brain tracer drainage, which may profoundly contribute to CSF accumulation observed in mice with DS.

### Lymphatic or systemic Piezo1 activation ameliorates disease phenotypes in mice with DS

**Fig. 8:**
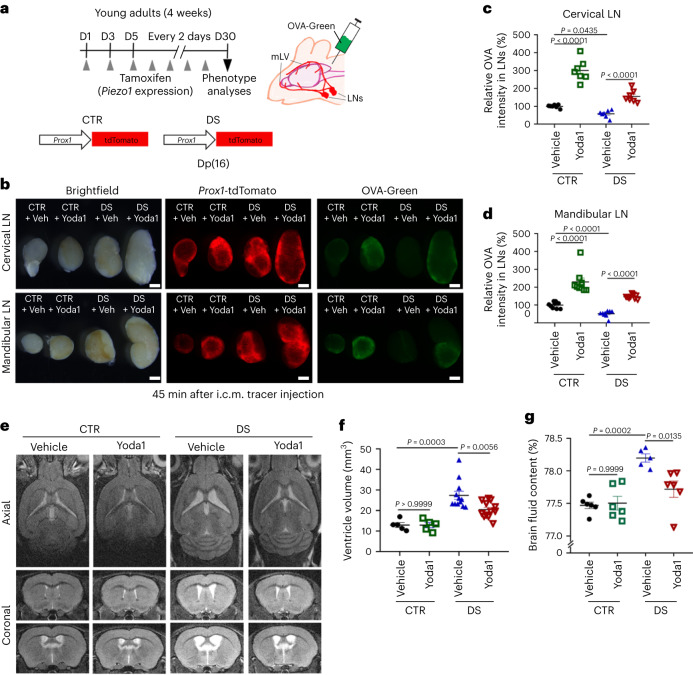
Systemic Yoda1 treatment enhances CSF drainage and reduces ventricle volume in a DS model. **a**, Experimental scheme. Young-adult control mice (*Prox1*-tdTomato) and mice with DS (*Prox1*-tdTomato; Dp(16)1Yey/+; 4 weeks old) were i.p. injected with vehicle or Yoda1 (213 µg per kg (body weight)) every 2 days for 30 days. **b**, The OVA-Green tracer was then i.c.m. injected, and, after 45 min, cervical and mandibular LNs were collected and imaged; scale bars, 500 µm. Longer-exposure images revealed a clear difference in tracer drainage amounts between control mice and mice with DS (Supplementary Fig. 6b). **c**,**d**, Relative fluorescence intensity of the tracer drained to the cervical (**c**) and mandibular (**d**) LNs. One data point represents the total fluorescence intensity of the left and right LNs of one mouse; *n* = 7–9 mice per group. Data were analyzed by one-way ANOVA (*P* < 0.0001) followed by a Tukey’s multiple comparison test (**c**) or a Kruskal–Wallis *H*-test (*P* < 0.0001) followed by a Bonferroni multiple testing correction (statistical significance, *P* < 0.0083; **d**). **e**,**f**, Brain magnetic resonance imaging was conducted (**e**), and ventricular volume was determined (**f**). Three-dimensional rendered images of the brain MRI are also shown (Extended Data Fig. 3). **g**, Brain fluid content was measured; *n* = 5–12 mice per group. Data were analyzed by Kruskal–Wallis *H-*test (*P* < 0.0001) followed by a Bonferroni multiple testing correction (statistical significance, *P* < 0.0083; **f**) or a one-way ANOVA (*P* < 0.0001) followed by a Bonferroni multiple testing correction (**g**). Data are presented as mean values ± s.e.m. Source data

We next studied the effect of lymphatic Piezo1 overexpression on excessive CSF deposits in mice with DS. To achieve this, we bred *Prox1*-tdTomato/*Piezo1*^TG_LEC^ mice with Dp(16) mice with DS^59^, resulting in four experimental groups: control, *Piezo1*^TG_LEC^, DS and DS/*Piezo1*^TG_LEC^, all carrying the *Prox1*-tdTomato allele (Fig. 7i). Lymphatic Piezo1 overexpression was induced by tamoxifen injection, and, after 7 weeks, the mice were subjected to brain fluid outflow tests. Remarkably, lymphatic Piezo1 overexpression for 7 weeks in the DS background dramatically improved impaired brain fluid drainage to the cervical and mandibular LNs (Fig. 7j–l and Supplementary Fig. 6a). In agreement with the enhancement in drainage, lymphatic Piezo1 activation significantly suppressed ventricular volume and pathological CSF buildup in mice with DS, as evidenced by brain MRI scan and brain fluid content assessment (Fig. 7m–o and Extended Data Fig. 3). Furthermore, we also assessed the therapeutic effect of Yoda1-mediated Piezo1 activation on CSF physiology in mice with DS. Yoda1 was i.p. injected into adult control wild-type or Dp(16) mice with DS every 2 days for 30 days, and the mice were evaluated for brain fluid drainage (Fig. 8a). Indeed, systemic Yoda1 treatment noticeably reversed diminished CSF outflow to the cervical and mandibular LNs in Dp(16) mice with DS (Fig. 8b–d and Supplementary Fig. 6b). In addition, Yoda1 i.p. administration also reduced the ventricular volume and brain fluid content observed in Dp(16) mice with DS (Fig. 8e–g and Extended Data Fig. 3). Together, these results demonstrate that lymphatic or pharmacological activation of Piezo1 can enhance CSF drainage and efficiently ameliorate ventricular enlargement in mice with DS.

## Discussion

In this study, we present a compelling body of experimental evidence that highlights the therapeutic potential of activating lymphatic mechanotransduction to enhance brain fluid drainage. Our investigation was centered on a hypothesis that Piezo1-mediated mechanotransduction within mLVs plays a key role in regulating CSF homeostasis and unveiled the essential role of Piezo1 in the development and function of mLVs. Furthermore, we demonstrated that activating Piezo1 can profoundly improve mLV function in facilitating CSF outflow and subsequently applied this innovative concept to two neurological disease models, identifying the potential therapeutic benefits of Piezo1 activation in alleviating excessive CSF accumulation observed in hydrocephalus and ventriculomegaly.

This study also revealed that blockage of brain fluid drainage through mLVs may serve as a major trigger for hydrocephalus in the kaolin-injected brain, which is characterized by a significant delay in tracer outflow. Importantly, both lymphatic Piezo1 overexpression and systemic Piezo1 stimulation with Yoda1 treatment comparably ameliorated reduced CSF outflow, ventricular enlargement and increased ICP (Figs. 2 and 3). As previously reported^4,7^, Piezo1 was also detected in other cell types, including blood vessel cells (Supplementary Fig. 7), which could have been activated by Yoda1 and may have contributed to the overall effects observed with Yoda1 treatment. Nonetheless, our investigation also unveiled that Yoda1 did not produce the same level of drainage improvement when lymphatic *Piezo1* was deleted in *Piezo1*^dLEC^ mice (Fig. 3i–k). This observation suggests that the enhanced outflow of brain fluid facilitated by Yoda1 primarily results from the activation of lymphatic mechanotransduction by Yoda1. Additionally, according to The Human Protein Atlas single-cell RNA database (as of September 2023), Piezo1 displays its highest expression levels in LECs, surpassing approximately 90 other distinct cell types. Collectively, these findings underscore that activating Piezo1 within lymphatic vessels through a transgenic or pharmacological approach is effective in boosting brain fluid drainage and that Piezo1 present in mLVs plays a crucial role in enhancing brain fluid outflow.

For Piezo1-activated fluid drainage, we propose two distinct mechanisms functioning at separate vascular sites: the enhancement of fluid absorption at the initial lymphatics and the improvement of lymphatic transport at the collecting lymphatics (Fig. 4). At the initial lymphatics, the removal of vascular endothelial-cadherin (VE-cadherin) from the cell membrane induced by Yoda1 could be a critical factor in enhancing the absorption of CSF and, consequently, its drainage from the brain. VE-cadherin is a widely recognized regulator of vascular permeability in both blood vessels^65,66^ and lymphatic vessels^49^. Moreover, VE-cadherin is specifically situated at the discontinuous ‘button-like’ junctions present in the initial lymphatics^48^. Its absence from these junctions has been linked to an augmentation in lymphatic absorption^67^. Our findings are consistent with the concept of VE-cadherin internalization from the membrane following phosphorylation, leading to enhanced fluid absorption. However, it is important to emphasize that the loss of VE-cadherin at the cell membrane does not necessarily indicate an increased fluid drainage rate. For instance, reduced VE-cadherin expression in the downstream collecting vessels could potentially cause increased lymphatic leakage, which often occurs in cases of dysfunctional lymphatics or lymphatics exposed to inflammatory conditions^49,67–69^. Therefore, it is imperative to assess the functional implications of VE-cadherin loss from the cell membrane through multiple experimental methods. Our observation of diminished VE-cadherin expression in cultured LECs following Yoda1 treatment is corroborated by functional assessments conducted through various in vitro, ex vivo and in vivo approaches. In addition, our study uncovered an additional mechanism contributing to Yoda1-induced improvement in fluid drainage. Yoda1 induces the dilation of lymphatic vessels and inhibits the contractility of collecting lymphatics. A previous study eloquently showed that lymphangions can switch from a pumping mode to a conduit mode when the outlet pressure drops below the inlet pressure and the contraction of postnodal lymphatic collectors hinders passive flow^51^. Therefore, it appears that Yoda1 promotes lymphatic transport by maximizing the conduit characteristics of the collectors through vessel dilation and a reduction in pumping activity. Nonetheless, this explanation is valid only under the premise that a higher fluid pressure exists upstream than downstream in the lymphangion chains in vivo. However, confirming this assumption in real time using Yoda1-treated mice presents substantial technical challenges. Furthermore, we should not disregard the potential involvement of other parameters that could play a role in Piezo1-mediated augmentation of fluid drainage. These factors may encompass the behaviors of intrinsic and extrinsic lymphatic valves (as elaborated below), the architectural features of lymphatic vessel walls, the characteristics of lymph flow in terms of pattern and volume and the shear stress experienced by lymphatic walls. Given that a multitude of physical, anatomical, structural and physiological variables collectively influence the ultimate outcome of lymphatic transport, further research is required to achieve a more comprehensive understanding of this process.

Our previous studies have underscored the critical role of Piezo1 in lymphatic vessel development^13,14^. Here, we extended our investigation to explore the significance of lymphatic valves in the outflow of brain fluids. We accomplished this by selectively targeting Piezo1 located within the valves of mLVs through the administration of a low dose of tamoxifen, which was achievable because the *Prox1*-CreER^T2^ allele, present in valvular LECs, displayed heightened sensitivity to tamoxifen compared to its counterpart in luminal LECs^14^. This increased sensitivity primarily stems from the elevated expression levels of Prox1 in valvular LECs^47^. Indeed, administering a low dose of tamoxifen to neonatal *Piezo1*^dLEC^ mice led to a notable reduction in the total number of valves in basal mLVs with no apparent alterations in the density or thickness of the mLVs present in the same regions. Notably, these mice still exhibited a reduction in brain fluid drainage (Extended Data Fig. 4a–g). When the same treatment regimen was applied to adult *Piezo1*^dLEC^ mice, it did not impact the integrity of their completely matured mLV valves. Apparently, this treatment regimen was ineffective for fully grown lymphatic valves. Nevertheless, a reduction in brain fluid outflow was still evident (Extended Data Fig. 4h–o), indicating that despite the absence of morphological changes, *Piezo1* deletion in the adult lymphatic valve can still impair brain fluid drainage, presumably due to a functional decline in mLV valves. Nonetheless, it remains possible that mosaic and/or partial *Piezo1* deletion in luminal LECs, in addition to valvular LECs, could occur with this low-dose tamoxifen regimen, potentially impairing brain fluid drainage. Together, these data highlight the key role of mLV valves in brain fluid drainage.

We successfully applied this concept of mechanoactivation of lymphatics to two neurological disease models, hydrocephalus and ventriculomegaly, which commonly present ventricular enlargement and excessive CSF buildup. We used the kaolin-induced hydrocephalus mouse model in this report (Figs. 5 and 6). A few days after the initiation of the hydrocephalus model, brain fluid outflow via mLVs was entirely halted. This was accompanied by a sixfold increase in ventricular volume and a fivefold surge in ICP. Consistent with these abrupt and severe changes, a noticeable reduction of physical activity also became evident in these mice. Our study revealed that the genetic activation of *Piezo1* enhanced CSF outflow even before the appearance of any signs of lymphatic growth (Extended Data Fig. 5), implying that the CSF drainage improvement was a result of immediate functional enhancement of mLVs (that is, lymphatic activation), not by the expansion of mLVs (that is, lymphangiogenesis). Therefore, despite the differences between kaolin-induced mouse hydrocephalus and human idiopathic NPH, our current study offers a robust conceptual justification and a technical foundation for exploring Yoda1 or its derivatives as potential treatments for idiopathic NPH.

However, ventriculomegaly refers to the abnormal expansion of the ventricular dimensions, often occurring without a noticeable increase in ICP^40^. It is frequently associated with brain atrophy or shrinkage, where enlargement of the ventricles can be a result of loss of brain tissue, leading to a compensatory increase in CSF volume. A variety of reasons, including neurodegenerative diseases, traumatic brain injury or infections, can cause the loss of brain tissue. However, like hydrocephalus, impaired CSF outflow can also cause ventriculomegaly in the absence of brain shrinkage. For a long time, ventriculomegaly in DS has been thought to be caused by defective neurogenesis and age-related brain shrinkage^41,45^. In this study, our data led us to propose an additional mechanism where disturbed development and/or dysfunction of mLVs may aggravate the onset and progress of ventriculomegaly in DS. We showed that mLVs of mice with DS are incompletely developed and abnormally formed (Fig. 7). Our discovery is supported by multiple previous reports showing disturbed lymphatic development in human fetuses and mouse embryos with DS^61–64^. These previous studies reported impaired LEC differentiation during early embryonic development that hinders the development of jugular lymphatic sacs, resulting in nuchal edema (translucency) in aneuploid fetuses. Nuchal edema, detected by prenatal ultrasound screenings, refers to tissue swelling at the back of the fetal neck that serves as a hallmark for chromosomal abnormalities during pregnancy. Although nuchal edema disappears after the first trimester, lymphatic malformation and dysfunction may persist throughout the life of an individual^70^. Thus, it is possible that disturbed postnatal mLV development in DS could lead to ventricular enlargement in the early stages of life. Alternatively, the pathological outcome of compromised lymphatics in DS may be manifested only in later stages of life because aging serves as the key risk factor underlying lymphatic function deterioration, as shown by previous studies^25,28^ and our current data (Fig. 3). Notably, our study revealed that Piezo1-mediated promotion of CSF drainage was more pronounced in older mice than in younger mice. Accordingly, aged mice with DS may be more responsive to the Piezo1 effect on activating CSF outflow and normalizing their ventricular volume. Together, our study demonstrates that Piezo1 activation could be a promising therapeutic modality to address excessive CSF accumulation in DS.

In sum, our study opens a new avenue toward treatment of abnormal accumulation of CSF and its associated symptoms. Our data provide a solid experimental foundation for the development of new drugs that may offer an alternative to highly invasive surgery for individuals with hydrocephalus or ventriculomegaly. Moreover, this therapeutic concept may be applicable to other neurological disorders that have been shown to respond to VEGF-C-induced mLV activation, such as Alzheimer’s disease, Parkinson’s disease and multiple sclerosis^26,27,29,33,34^. Exploring the therapeutic benefits of genetic and pharmacological activation of Piezo1 in animal models of these diseases would be a valuable future pursuit.

## Methods

### Study approval

All mouse experiments were approved by the University of Southern California Institutional Animal Care and Use Committee (principal investigator Y.-K.H.) and the University of South Florida Institutional Animal Care and Use Committee (principal investigator J.S.).

### Mice and related reagents

Mice were housed in air-filtered clear cages under a 12-h light/12-h dark cycle and were fed ad libitum. Mice were obtained from the following sources: C57BL/6J (The Jackson Laboratory); *Prox1-*eGFP^47^ and *Prox1-*tdTomato^46^ (Mutant Mouse Resource and Research Centers); *ROSA26-*LSL-tdTomato (The Jackson Laboratory); *Prox1*-CreER^T2^ (a gift from T. Mäkinen, Uppsala University)^71^; *Piezo1*^fl/fl^ (*Piezo1*^tm2.1Apat^/J; The Jackson Laboratory)^72^; *Piezo1*-transgenic line (CAG-LSL-*Piezo1*; previously generated and reported by the authors)^13^ and Dp(16)1Yey/+ (B6.129S7-Dp(16Lipi-Zbtb21)1Yey/J, The Jackson Laboratory)^59,73^. The absence of the potential toxicity of Cre protein expression was confirmed by comparing lymphatic vessel density and thickness as well as drainage functionality of wild-type and *Prox1*-CreER^T2^ mice that do not carry *Piezo1* floxed alleles (Supplementary Fig. 3). Lymphatic *Piezo1*-knockout mice (*Piezo1*^dLEC^) have *Prox1*-CreER^T2^ and *Piezo1*^fl/fl^ alleles with or without a lymphatic reporter allele (*Prox1-*tdTomato or *Prox1-e*GFP). Lymphatic Piezo1 overexpression mice (*Piezo1*^TG_LEC^) harbor *Prox1*-CreER^T2^ and *Piezo1*-transgenic alleles (CAG-LSL-*Piezo1*) with or without a lymphatic reporter allele. DS and *Piezo1*^TG_LEC^ compound mice have *Prox1-*tdTomato, Dp(16)1Yey/+, *Prox1-*CreER^T2^ and *Piezo1*^TG^ alleles. Males and females were nonselectively used for each experiment as we did not find sex-specific differences in phenotype. Tamoxifen (MP Biomedicals) was dissolved in dimethyl sulfoxide (DMSO) and diluted in sunflower seed oil (1:9 (vol/vol) DMSO:sunflower seed oil). Diluted tamoxifen was injected into pups (once) and adults (twice) at 50 mg per kg (body weight) per day to induce Cre recombinase activity. Yoda1 (Sigma-Aldrich) was dissolved in DMSO (710 µg ml^–1^) and diluted in phosphate-buffered saline (PBS; 3:100 (vol/vol) DMSO:PBS) before injection at a final concentration of 213 μg per kg (body weight) per day, which was previously established^13,14^. Supplementary Table 1 provides the strain, sex, number and age information of all mice used in each experiment. Mice of the indicated ages in Supplementary Table 1 were randomly selected for each experiment.

### Cell culture-related assays

Human dLECs were isolated from deidentified human foreskin samples and cultured as previously described^74,75^ with approval by the University of Southern California Institutional Review Board (principal investigator Y.-K.H.). LECs lower than six to seven population passages were used for all experiments. Yoda1 was dissolved in DMSO (2 mM) and added to cell culture medium at a final concentration of 0.3–2 µM. For siRNA transfection, LECs were seeded in T75 flasks and incubated for 24 h. Control siRNA or Piezo1 siRNA (Dharmacon, L-020870-03-0005) was mixed with Lipofectamine RNAiMAX transfection reagent (Invitrogen, 13778150). The siRNA–reagent complex was added to the cell culture 24 h before performing western blotting or drainage tests using a lymphatics-on-chip (see below). A cell death detection enzyme-linked immunosorbent assay (ELISA) kit (Roche Molecular Biochemicals, 11544675001) was used to evaluate the potential cytotoxicity of Yoda1. LECs were seeded on six-well plates and incubated for 24 h. The cells were treated with 0.5, 1 or 2 μM Yoda1 or vehicle (DMSO) for 8 h before the cell death assay. For cell proliferation assays, LECs were seeded and incubated for 24 h and treated with vehicle, Yoda1 or Yoda1 + inhibitor (l-NAME (Selleckchem, S2877), capivasertib (Selleckchem, S8019), axitinib (Selleckchem, S1005), SAR131675 (Selleckchem, S2842) and cabozantinib malate (Selleckchem, S4001)). After 2 days, the cells were detached and counted with a Cellometer Auto T4 (Nexcelom Bioscience).

### Western blotting

Western blotting assays were performed as described previously^13^. Antibodies were acquired from the following sources: β-actin (Sigma-Aldrich, A5441), p-CDH5 (Try 658; Thermo Fisher Scientific, 44-1144G), p-CDH5 (Try 685; ECM Biosciences, CP1981), CDH5 (Santa Cruz Biotechnology, sc-6458), p-AKT(Ser 473; Cell Signaling Technology, 4060), AKT (Cell Signaling Technology, 9272), eNOS (Cell Signaling Technology, 32027), p-eNOS (Ser 1177; Cell Signaling Technology, 9571), VEGFR2 (R&D Systems, AF357), p-VEGFR2 (Tyr 1054/Tyr 1059; Invitrogen, 44-1047G), VEGFR3 (Santa Cruz Biotechnology, sc-321), p-VEGFR3 (Tyr 1230/Tyr 1231; Cell Applications, CY1115), Prox1 (DSHB, AB2619013) and LYVE1 (R&D Systems, AF2089). Rabbit polyclonal anti-Piezo1 was generated by the authors (GenScript).

### Immunofluorescence staining

mLVs from *Prox1-*tdTomato, *Prox1-*eGFP, *Piezo1*^TG_LEC^ and *Piezo1*^dLEC^ mice were stained using anti-LYVE1 (Angiobio, 11-034), anti-Pdpn (DSHB, AB531893), anti-VEGFR3 (R&D Systems, AF743) and/or anti-Piezo1 (generated by the authors) by following the standard whole-mount immunostaining protocol or frozen section immunostaining protocol^12,13,75^. Specificity of rabbit anti-Piezo1 generated by the authors was verified using meningeal tissues from wild-type, *Piezo1*^TG_LEC^ and *Piezo1*^dLEC^ mice as well as western blotting analyses (Extended Data Fig. 1f and Supplementary Fig. 2). Alexa Fluor fluorescent secondary antibodies (Invitrogen, A21206, A21113, A21207 and A11058) were used to detect protein signal. Fluorescence images were captured with a Zeiss LSM 800 AxioObserver.M2 confocal microscope system or a Zeiss Apotome microscope (AxioZoom V16) controlled by Zen 2.6 software. Randomly selected images were used for statistical analysis.

### RNAscope

The RNAscope analysis was performed using an RNAscope Multiplex Fluorescent Reagent kit v2 with TSA Vivid Dyes (Advanced Cell Diagnostics, 323270) and following the manual (Advanced Cell Diagnostics, UM323100) provided by Advanced Cell Diagnostics.

#### Preparation and pretreatment of samples

Control, *Piezo1*^TG_LEC^ and *Piezo1*^dLEC^ mice were i.p. injected with tamoxifen (MP Biomedicals; 50 mg per kg (body weight) twice, 3 days apart) at the age of 6 weeks. After 6 days from the first tamoxifen administration, skulls were collected and fixed in 4% (wt/vol) paraformaldehyde (PFA; Sigma, 252549-1L) at 4 °C for 1 day. The meninges were isolated from the bond and frozen in Tissue-Tek optimum cutting temperature medium (VWR, 25608-930). Frozen blocks were sectioned by cutting 10-μm-thick sections to mount on Superfrost Plus slides (VWR, 48311-703) before air drying for 2 h at –20 °C. The slides were washed with PBS, baked for 30 min at 60 °C in a HybEZ II Hybridization System (Advanced Cell Diagnostics, 321721) and postfixed with 4% (wt/vol) PFA (Sigma, 252549-1L) at 4 °C for 15 min. The tissues were serially dehydrated with ethanol, and RNAscope hydrogen peroxide was applied to the samples for 10 min at room temperature before the samples were boiled in RNAscope 1× Target Retrieval Reagent. RNAscope Protease III was added to the samples after creating a barrier around the tissue section on the slide with an ImmEdge hydrophobic barrier pen (Vector Laboratory, H-4000). The samples were then incubated for 30 min at 40 °C in a HybEZ II Hybridization System.

#### RNAscope fluorescent assay and imaging

The RNAscope probes Mm-Piezo1 (Advanced Cell Diagnostics, 400181) and Mm-Prox1-C2 (Advanced Cell Diagnostics, 488591-C2) were mixed, added to the samples and incubated for 2 h at 40 °C in a HybEZ II Hybridization System. RNAscope 3-plex Positive Control Probe (Advanced Cell Diagnostics, 320881) and RNAscope 3-plex Negative Control Probe (Advanced Cell Diagnostics, 320871) were used as the controls for the RNAscope reactions, respectively. RNAscope Multiplex FL v2 AMP1 was applied to the samples and hybridized by incubating for 30 min at 40 °C in a HybEZ II Hybridization System. In the same way, RNAscope Multiplex FL v2 AMP2 and AMP3 were hybridized sequentially. To develop horseradish peroxidase (HRP) channel signal for Piezo1 mRNA, RNAscope Multiplex FL v2 HRP-C1 was added to the samples before incubation for 15 min at 40 °C in a HybEZ II Hybridization System. TSA Vivid Fluorophore 520 was then applied and incubated for 30 min at 40 °C in a HybEZ II Hybridization System. The reaction was terminated by adding RNAscope Multiplex FL v2 HRP blocker. Similarly, RNAscope Multiplex FL v2 HRP-C1 and TSA Vivid Fluorophore 570 were applied to develop HRP channel signal for *Prox1* mRNA. To label the nuclei, DAPI was added to the samples before incubation for 30 s at room temperature. ProLong Gold Antifade Mountant (Thermo Fisher Scientific, P36930) was added on the slides before a coverslip was placed over the tissue. Fluorescence images were captured with a Zeiss Apotome microscope (AxioZoom V16). For statistical analysis, 16 of the images acquired from three to five mice were randomly selected.

### VEGF-C and VEGF-D ELISA

Adult wild-type mice were i.p. injected with vehicle or Yoda1 (213 μg per kg (body weight)) at 18 h and 90 min before collecting the brains. *Piezo1*^TG_LEC^ mice were i.p. injected with tamoxifen (50 mg per kg (body weight) twice, 3 days apart), and the brains were collected after 6 days from the first injection. Brains were homogenized in PBS (1 ml PBS per 100 mg of brain tissue), and whole-brain extracts were prepared. Concentrations of VEGF-C and VEGF-D in the brain extracts were quantified using a mouse VEGF-C ELISA kit and a mouse VEGF-D ELISA kit (CUSABIO, CSB-E07361m and CSB-E07357m, respectively).

### i.c.m. injection and imaging

Tracers were injected into the cisterna magna as described previously^28,76^. Mice were anesthetized with isoflurane (Kent Scientific Corporation), and the head was tightly placed with the head adaptors of a stereotaxic instrument (World Precision Instruments, 505213). A surgical incision was made to separate the subcutaneous tissue and muscles of the nuchal. Mice were placed so that the head formed a nearly 135° angle with the body to expose the dura mater on the cisterna magna. The following fluorescent tracers were used: ALB-Red (0.5 mg ml^–1^, Molecular Probes, A13101), OVA-Green (0.5 mg ml^–1^, Molecular Probes, 034781) and ICG (2.5 mg ml^–1^, MP Biomedicals, 155020). Tracers were loaded in a syringe (World Precision Instruments, NANOFIL) that was connected with a 33-gauge needle (World Precision Instruments, NF33BV-2) and Microinjection Syringe Pump system (World Precision Instruments, UMP3T-1) and injected (3.5 μl) into the cisterna magna at a rate of 2.5 µl min^–1^. The incision was immediately sutured. For ICG time-lapse imaging, the tracer was i.c.m. injected as described earlier into *Prox1*-eGFP mice. The mandibular skin was surgically removed for imaging under a stereomicroscope. The green fluorescence images were first taken to reveal the location of LNs, and near-infrared fluorescence images were obtained every 5 min to record the arrival of ICG to the mandibular LNs. For the brain tracer drainage experiments, cervical and mandibular LNs were surgically collected and imaged for the fluorescent tracer signals at 45–60 min after injection. Images were captured with a Leica stereomicroscope (Leica, M165 FC) controlled by Leica Application Suite X 3.7.6 software, and tracer intensity was measured using ImageJ software (National Institutes of Health). The relative amount of outflowed tracer was quantified by combining the tracer intensities in LNs on both sides.

#### Mouse hydrocephalus model

The mouse hydrocephalus model was induced as previously described^76^. A sterile solution of 15% kaolin (suspended in PBS; Sigma-Aldrich, 795453) was loaded in a syringe (Hamilton, 80201) connected with a sterile 31-gauge needle. Under a dissection microscope, the needle was moved into the cisterna magna 3–5 mm deep from the skin, and 10 μl of kaolin was injected using a Microinjection Syringe Pump system (World Precision Instruments, UMP3T-1). The muscles and skin incision were sutured (Redilene, P8698-SP), and mice were monitored over several days for recovery and health.

#### ICP measurement

ICP measurements were performed as previously described^77^ with minor modifications. Mice were anesthetized, and an incision was made as described in i.c.m. injection and imaging. A sterile 31-gauge needle connected with a pressure monitor (SYS-BP1, World Precision Instruments) was inserted into the cisterna magna under a dissection microscope. The needle was held for 1 min until an ICP value was obtained.

### MRI

Mice were anesthetized using 2% isoflurane in 90% oxygen delivered through a precision vaporizer (Kent Scientific Corporation) and transferred to a 7-T, 24-cm-bore horizontal magnetic resonance scanner (MR Solutions). Two-dimensional fast spin echo (FSE) T2-weighted magnetic resonance images were acquired in coronal and transverse orientations to identify anatomy. The transverse FSE T2-weighted imaging parameters were repetition time = 4,500 ms, echo time = 45 ms, number of averages = 3, echo train length = 7, slice thickness = 0.40 mm, field of view = 16 mm × 16 mm, matrix size = 256 × 238, in-plane resolution = 0.0625 × 0.0672 mm^2^ per pixel and number of slices = 32. The coronal FSE T2-weighted images had the following parameters: repetition time = 4,500 ms, echo time = 45 ms, number of averages = 3, echo train length = 7, slice thickness = 0.40 mm, field of view = 18 mm × 18 mm, matrix size = 238 × 256, in-plane resolution = 0.0756 × 0.0703 mm^2^ per pixel and number of slices = 32. Vital signs (respiration in breaths per min) were continuously monitored using a pneumatic pillow (SAII) placed over the abdominal region of the mouse. Body temperature was monitored using a rectal temperature probe (SAII) and maintained at 37 °C using a heated animal holder with a temperature controller unit (Minerve). Brain ventricle volumes were analyzed for all magnetic resonance images by 3D rendering using Multi-image Analysis GUI (Mango)^78^.

### Transcardiac perfusion

Transcardiac perfusion was performed following a previously described method^79^. Hydrocephalus was induced by i.c.m. injection of kaolin 5 days before transcardiac perfusion. Mice were anesthetized with a combination of ketamine (100 mg per kg (body weight), i.p.) and xylazine (20 mg ml^–1^, i.p.), and the heart was fully exposed by incision of the skin, diaphragm and ribs. An infusion 25-gauge needle (Terumo, SV25BLK) was inserted into the left ventricle, and a small incision was made in the right atrium. PBS containing heparin (10 U ml^–1^, final) was infused into the heart at a constant speed of 150 μl s^–1^ until the fluid outflow was clear of blood, and the liver turned pale. Importantly, our initial examination did not find significant differences in brain fluid content with versus without transcardiac perfusion with PBS before brain collection (Supplementary Fig. 8). We thus did not routinely perform transcardiac perfusion for brain fluid content measurement.

### Brain fluid content measurement

Brain fluid content was measured following a previously described method^80^ with minor modifications. The skin and skull were removed after decapitation of the mouse. The brain was isolated and weighed immediately (wet weight). For dehydration, the brain was placed on a hot plate at 110 °C for 24 h and reweighed (dry weight). Fluid content in the brain was calculated according to the following equation:

$${{\mathrm{Fluid}}}\,{{\mathrm{content}}}\,\left( \% \right)=\frac{{{\mathrm{wet}}}\,{{\mathrm{weight}}}-{{\mathrm{dry}}}\,{{\mathrm{weight}}}}{{{\mathrm{wet}}}\,{{\mathrm{weight}}}}\,\times\,100$$

### Lymphatic drainage test in PDMS chips

A lymphatics-on-chip was microfabricated as previously described^50,81–83^. Briefly, the PDMS chip was assembled by bonding a PDMS chip on top of a glass coverslip. After treatment with poly-l-lysine and glutaraldehyde, the PDMS chip was injected with 2.5 mg ml^–1^ rat tail collagen 1 (Corning) to surround two 250-μm acupuncture needles (Hwato). After the collagen polymerized, acupuncture needles were removed, and primary human dLECs were seeded into one of the channels, while the other channel was left as an acellular channel. To measure lymphatic drainage in the lymphatics-on-chip, 300 μl and 20 μl of cell culture medium was added to the acellular and lymphatic channels, respectively. The volume of medium in the two channels was measured after 12 h, and the drainage score was calculated according to the following equation:

$${{\mathrm{score}}}=\frac{{V}_{{{\mathrm{LEC}}}_1}}{({V}_{{{\mathrm{LEC}}}_0}+{V}_{{{\mathrm{Accell}}}_0})/2},$$

where ${V}_{{{\mathrm{LEC}}}_0}$ and ${V}_{{{\mathrm{LEC}}}_1}$ are the volumes of medium in the lymphatic channel before and after 12 h, respectively, and ${V}_{{{\mathrm{Accell}}}_0}$ is the volume in the acellular channel before 12 h. After drainage score measurement, the lymphatics-on-chips were stained as previously described^82^. Briefly, the chips were fixed with 4% PFA, permeated with 0.3% PBST (0.3% Triton X-100 in phosphate-buffered saline) and blocked with 3% bovine serum albumin overnight at 4 °C on a shaking platform. Anti-VE-cadherin (F11, Santa Cruz Biotechnology; 1:100) in blocking buffer was added and incubated overnight at 4 °C. Primary antibodies were washed overnight using PBS at 4 °C. Secondary antibody (Invitrogen, 1:500), phalloidin (actin, 1:200) and DAPI (Millipore Sigma, 1:500) were subsequently incubated in blocking buffer overnight at 4 °C in the dark. The chips were washed to remove fluorescent background before confocal microscopy. Confocal images were acquired with a Leica SP8 confocal microscope. Image quantification was performed with ImageJ software^84^.

### Excised lymphatic collector physiology assays

Supplemental Videos 1–6 show changes in the contractile parameters of an excised collecting lymphatic after exposure to vehicle, Yoda1 and Yoda1 + l-NAME.

#### Vessel isolation and cannulation

Mice were anesthetized with a combination of ketamine (100 mg per kg (body weight), i.p.) and inactin (100 mg per kg (body weight), i.p.) and placed on a plexiglass board next to a pillar made from Sylgard 184 (Dow Corning). The inguinal–axillary lymphatic vessel was exposed by making a 2-cm-long midline incision extended dorsally with two more incisions at the shoulder and groin to create a rectangular skin flap. The skin flap was gently pulled away from the body and pinned into the Sylgard pillar. Connective tissue was gently dissected to enable the skin to be pulled further, revealing both the inguinal and axillary LNs. The inguinal–axillary vessel was identified as the collecting lymphatic vessel connecting these two LNs, as previously reported^85^. Using fine microscissors, the axillary collecting lymphatic vessel was carefully excised along with adipose and connective tissues and transferred to a custom chamber coated with Sylgard 170 (Dow Corning) and containing Krebs buffer. The axillary vessel was then pinned to the Sylgard layer with 40-µm stainless steel wire. Adipose and connective tissues were removed from the vessel by careful microdissection at room temperature. The cleaned axillary lymphatic vessel was transferred to a 3-ml acrylic chamber filled with Krebs buffer that was mounted onto a custom breadboard (Thorlabs) onto which a pair of micromanipulators (LBM-7, Scientifica) was mounted. The micromanipulators were used to position two micropipettes (80 µm) at each end of the 3-ml chamber. The axillary lymphatic vessel was then cannulated on each micropipette and secured with a single knot of unbraided 4-0 silk suture.

#### Pressure system and diameter tracking

Once cannulated, the isolated vessel board was transferred to an inverted microscope (Observer Z1, Zeiss) and connected to a custom pressure control system. The pressure was controlled using LabView software that ran a pressure pump connected to the vessel via polyethylene tubing. The pressure was constantly measured at each end of the vessel by low-pressure transducers. The vessel was allowed to equilibrate to 37 °C for 1 h at a pressure of 2–3 cmH_2_O, while the temperature was controlled by a heat exchanger pumping warm water into the water jacket of the 3-ml acrylic chamber. The pressure transducer signals were amplified and recorded by a custom-written LabView program^86^. The same LabView program tracked and recorded the inner diameter of the vessels from a video image obtained by a FireWire camera (Basler) at 30 Hz.

#### Isolated vessel protocol

Isolated and cannulated collecting lymphatic vessels from the axillary region of mice were subjected to the following protocol to evaluate the actions of Yoda1 on lymphatic endothelium. To enable pairwise comparisons, collecting lymphatic vessels were allowed to contract spontaneously in the same vehicle used for diluting Yoda1 for vehicle control while the diameter was recorded. Pressure at each end of the vessel stepped from 0.5 cmH_2_O to 1, 2, 3, 5, 7 and 10 cmH_2_O. At the end of the pressure steps, the pressure was lowered to 3 cmH_2_O before adding Yoda1. Yoda1 (1 µM) was then added to the superfusion bath solution outside the vessel and incubated for 30 min before repeating the same pressure steps from 0.5 to 10 cmH_2_O. Finally, l-NAME (100 µM) and Yoda1 were combined and added to the bath for a 30-min incubation period at 3 cmH_2_O. After the incubation, the same pressure steps were repeated. At the end of all experiments, the superfusion buffer was replaced with a Ca^2+^-free Krebs buffer for 30 min. The intraluminal pressure was then lowered to 0.1 cmH_2_O, and the pressure was stepped to 0.5, 1, 2, 3, 5, 7 and 10 cmH_2_O to obtain the maximal vessel diameter at each pressure to calculate the basal tone.

#### Solutions and chemicals

Krebs buffer contained the following components: 141.4 mM NaCl, 4.7 mM KCl, 2 mM CaCl_2_•2H_2_O, 1.2 mM MgSO_4_, 1.2 mM NaH_2_PO_4_•H_2_O, 3 mM NaHCO_3_, 1.5 mM NaHEPES, 5 mM d-glucose and 0.1% bovine serum albumin (pH 7.4 at 37 °C). The Krebs buffer was sterile filtered and used within 1 week. This buffer was used for all dissection, cannulation and superfusion solutions. During the experiment, the superfusate was gradually supplemented by the continuous addition of Krebs buffer at a rate of 0.5 ml min^–1^. The Ca^2+^-free Krebs buffer was identical, except that CaCl_2_ was replaced with 3 mM EGTA. All chemicals except for bovine serum albumin (Affymetrix) were purchased from Sigma. l-NAME was dissolved in Krebs buffer at 100 mM and stored at –20 °C until the day of use.

#### Data analysis

After the experiments, separate custom-written LabView programs were used to detect and record the end-diastolic diameter, end-systolic diameter and contraction frequency. Data were copied into a Microsoft Excel file that was used to calculate the following contractile function parameters: amplitude = EDD – ESD and percent tone = [(max_D_ – EDD)/max_D_] × 100, where EDD is the end-diastolic diameter, ESD is the end-systolic diameter, and max_D_ is the maximal passive diameter at each pressure, which was obtained during the Ca^2+^-free portion of the experiment.

### Physical activity analyses

The open field test was performed following a published protocol^87^ with minor modifications. Mice were carried to a testing room isolated from sound and unintentional interruptions at least 1 h before the test. Mice were then placed in the center of an opaque white plastic open field chamber (45 cm × 25 cm), and free and uninterrupted movement of each mouse was recorded for 5 min. Using video tracking software (Tracker 6.0.10)^88^, the movement of the mouse was traced, and total distance, velocity and time spent in the center (27 cm × 5 cm) were quantified. The rotarod test was performed following a published protocol^89^ with minor modifications. Mice were placed on a rotarod cylinder (Ajanta-96, AJANTA) 25 mm in diameter rotating at 4 rpm. The rotational speed was gradually increased to 20 rpm min^–1^ until 40 rpm (maximum rotational speed) was reached. The mice were trained twice on the day before the experiment and on the morning of the experimental day to reduce variability between animals. The length of time the mouse continuously walked forward to keep from falling off the rotating rod was measured to determine the ability to maintain balance. The mesh hanging test was performed as previously described. We generated a mesh screen by cutting a 30-cm square of wire mesh (1-cm squares of 1-mm-diameter wire). Mice were placed in the center of the mesh screen, and the screen was rotated to an inverted position while starting the stopwatch. The screen was steadily held at the height of 50 cm above a pad until the mouse fell off (up to 3 min).

### Statistical analysis

Sample size was predicted and chosen by power analysis using G*Power (hhu). Parameters for the analysis were effect size (Cohen’s *d* or Cohen’s *f*) = 0.8–2, *α* error probability (significance level) = 0.05, power (1 – β error probability) = 0.8 and allocation ratio *N*_2_/*N*_1_ = 1. Effect sizes were calculated based on pilot experiments. The experiments were randomized. Data collection and analysis were not performed blind to the conditions of the experiment, but no animals or data points were excluded. Normality of the data was tested by Shapiro–Wilk or Kolmogorov–Smirnov test, and homoscedasticity was tested by Levene’s test or Box’s *M*-test. Sphericity was tested by Mauchly’s sphericity test, and the *F* critical value was adjusted by Greenhouse–Geisser correction. Parametric or nonparametric statistics were selected based on the result of the tests. A two-tailed, unpaired Student’s *t*-test or Mann–Whitney *U*-test was used to determine the statistical significance between two groups. A one-way ANOVA or Kruskal–Wallis *H*-test was performed to compare differences between multiple groups. A two-way repeated measures ANOVA was used to compare the time or pressure series data between two or three groups. Post hoc tests following one-way ANOVAs and two-way repeated measures ANOVAs were performed by Tukey’s multiple comparison test, Bonferroni’s multiple comparison test or Dunnett’s multiple comparison test, and statistical significance (*P*) was set at less than 0.05. The Bonferroni correction method was used as a post hoc test for the Kruskal–Wallis *H*-test. Statistical significance of the Bonferroni correction method was determined based on the number of groups and is indicated in the figure legends. Statistical analyses were performed using SPSS 12 (IBM) and GraphPad Prism 8 (GraphPad Software).

### Reporting summary

Further information on research design is available in the Nature Portfolio Reporting Summary linked to this article.

## Online content

Any methods, additional references, Nature Portfolio reporting summaries, source data, extended data, supplementary information, acknowledgements, peer review information; details of author contributions and competing interests; and statements of data and code availability are available at 10.1038/s41593-024-01604-8.

## Supplementary information

Supplementary Information Supplementary Figs. 1–8 and unprocessed western blot for Supplementary Fig. 5a.

Reporting Summary

Supplementary Table 1 Species, strain, sex, number and age of animals in every experiment.

Supplementary Data 1 Source data for the Supplementary figures.

Supplementary Video 1 Video of the contractile activity of a collecting lymphatic vessel from the flank skin connecting the axillary and inguinal LNs. The vessel was surgically isolated from a mouse, cannulated on glass pipettes and held at a pressure of 3 cmH_2_O for all subsequent videos. The vessel was bathed in Krebs buffer in this ~2-min video.

Supplementary Video 2 Video of the contractile activity of the same axillary collecting lymphatic vessel from Supplementary Video 1 right after the superfusion of Krebs buffer containing 2 µM Yoda1.

Supplementary Video 3 Video of the contractile activity of the same axillary collecting lymphatic vessel from Supplementary Video 2 but after 5 min of superfusion of Krebs buffer containing 2 µM Yoda1 to demonstrate steady-state effects.

Supplementary Video 4 Video of the contractile activity of the same axillary collecting lymphatic vessel from Supplementary Video 3 after superfusion of Krebs buffer containing 100 µM l-NAME for 20 min.

Supplementary Video 5 Video of the contractile activity of the same axillary collecting lymphatic vessel from Supplementary Video 4 immediately after superfusion of Krebs buffer containing both 2 µM Yoda1 and 100 µM l-NAME.

Supplementary Video 6 Video of the contractile activity of the same axillary collecting lymphatic vessel from Supplementary Video 5 but after 5 min of superfusing Krebs buffer containing both 2 µM Yoda1 and 100 µM l-NAME to demonstrate steady-state effects.

## Source data

Source Data Fig. 1 Statistical source data.

Source Data Fig. 2 Statistical source data.

Source Data Fig. 3 Statistical source data.

Source Data Fig. 4 Statistical source data.

Source Data Fig. 4 Unprocessed blots.

Source Data Fig. 5 Statistical source data.

Source Data Fig. 6 Statistical source data.

Source Data Fig. 7 Statistical source data.

Source Data Fig. 8 Statistical source data.

Source Data Extended Data Fig. 1 Statistical source data.

Source Data Extended Data Fig. 1 Unprocessed blots.

Source Data Extended Data Fig. 2 Statistical source data.

Source Data Extended Data Fig. 4 Statistical source data.

Source Data Extended Data Fig. 5 Statistical source data.

Source Data Extended Data Fig. 7 Statistical source data.

Source Data Extended Data Fig. 8 Statistical source data.

Source Data Extended Data Fig. 8 Unprocessed blots.

Source Data Extended Data Fig. 9 Statistical source data.

Source Data Extended Data Fig. 9 Unprocessed blots.

Source Data Extended Data Fig. 10 Statistical source data.

## Data Availability

All data supporting the findings in this study are available within the article and its Supplementary Information. Source data are provided with this paper.
