# Publisher Correction: Piezo1 regulates meningeal lymphatic vessel drainage and alleviates excessive CSF accumulation

**DOI:** 10.1038/s41593-025-02176-x

**Published:** 2025-11-18

**Authors:** Dongwon Choi, Eunkyung Park, Joshua Choi, Renhao Lu, Jin Suh Yu, Chiyoon Kim, Luping Zhao, James Yu, Brandon Nakashima, Sunju Lee, Dhruv Singhal, Joshua P. Scallan, Bin Zhou, Chester J. Koh, Esak Lee, Young-Kwon Hong

**Affiliations:** 1 Department of Surgery, Norris Comprehensive Cancer Center, Keck School of Medicine, University of Southern California, Los Angeles, CA USA; 2 Department of Biochemistry and Molecular Medicine, Norris Comprehensive Cancer Center, Keck School of Medicine, University of Southern California, Los Angeles, CA USA; 3 Nancy E. and Peter C. Meinig School of Biomedical Engineering, Cornell University, Ithaca, NY USA; 4 Division of Plastic and Reconstructive Surgery, Beth Israel Deaconess Medical Center, Harvard Medical School, Boston, MA USA; 5 Department of Molecular Pharmacology and Physiology, University of South Florida, Tampa, FL USA; 6 New Cornerstone Science Laboratory, State Key Laboratory of Cell Biology, Shanghai Institute of Biochemistry and Cell Biology, Center for Excellence in Molecular Cell Science, Chinese Academy of Sciences, University of Chinese Academy of Sciences, Shanghai, China; 7 Division of Pediatric Urology, Texas Children’s Hospital, Baylor College of Medicine, Houston, TX USA

**Keywords:** Developmental disorders, Hydrocephalus, Cardiovascular biology, Brain

Correction to: *Nature Neuroscience* 10.1038/s41593-024-01604-8, published online 25 March 2024.

In the version of the article initially published, the *y* axis of Fig. 5h read “0, 25, 25, 25, 25, 25, 30” and has now been corrected to “0, 5, 10, 15, 20, 25, 30” in the HTML and PDF versions of the article.

