# Supplementary material for: Piezo1 regulates meningeal lymphatic vessel drainage and alleviates excessive CSF accumulation
Source: Nat Neurosci. 2024 Mar 25;27(5):913–26. doi: 10.1038/s41593-024-01604-8 (PMC11088999; doi:10.1038/s41593-024-01604-8)
Supplement: Supplementary file 1 — Supplementary Figs. 1–8 and unprocessed western blot for Supplementary Fig. 5a. [file 41593_2024_1604_MOESM1_ESM.pdf]

nature neuroscience

Article https://doi.org/10.1038/s41593-024-01604-8

Piezo1 regulates meningeal lymphatic vessel drainage and alleviates excessive CSF accumulation

In the format provided by the authors and unedited

SUPPLEMENTAL FIGURES

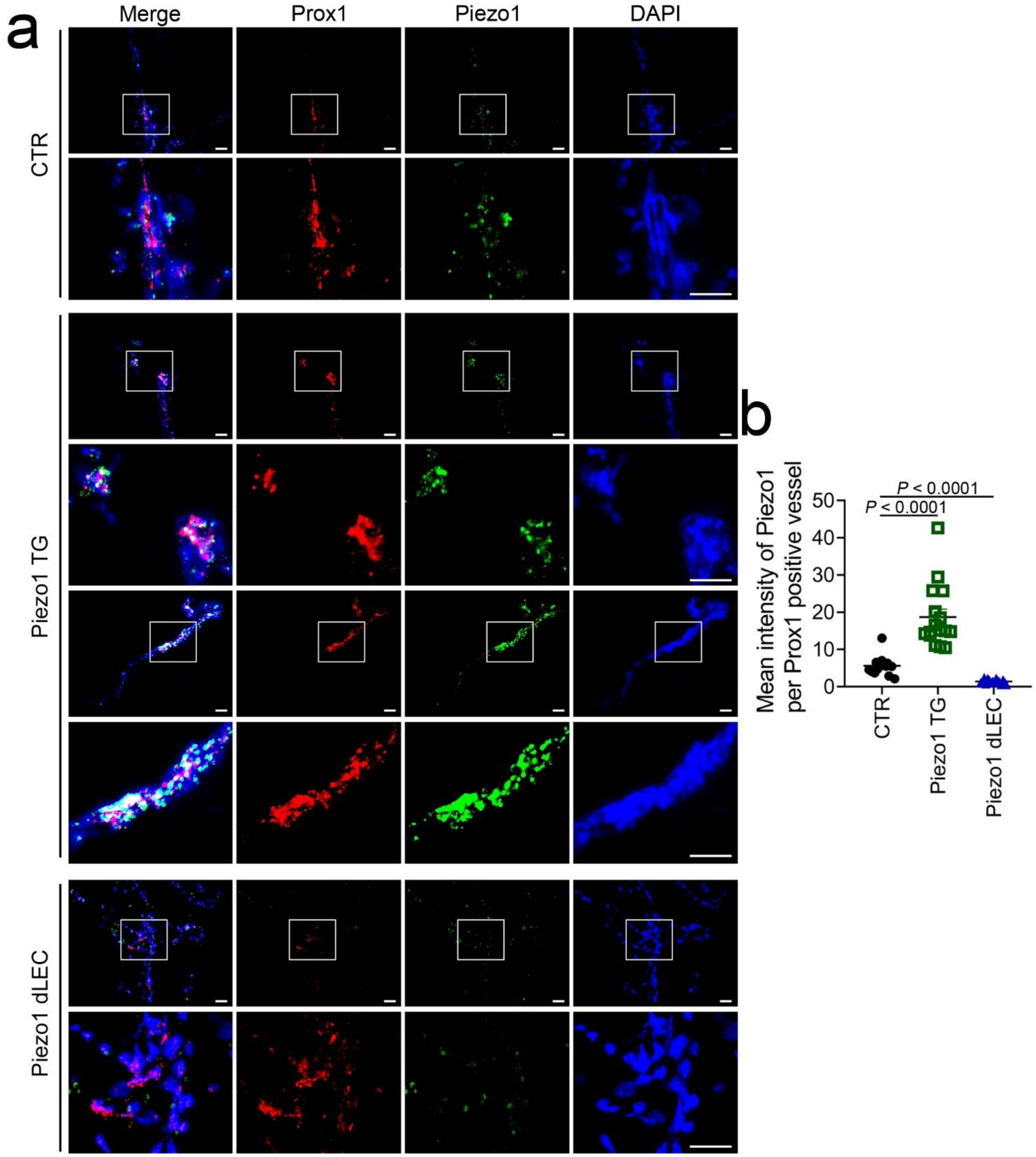

Supplementary Fig. 1 | Expression of Piezo1 mRNA in Mouse Meningeal Lymphatic Vessels. (**a**) Wild-type control (CTR), Piezo1TG_LEC, and Piezo1dLEC mice (6-8 weeks old mice) received two i.p. injections of Tamoxifen (50 mg/kg) three days apart. The meninges were isolated 6 days after tamoxifen injection and their frozen sections were prepared. Detection of Piezo1 mRNA in mLVs of wild-type control (CTR), Piezo1^{TG_LEC}, and Piezo1^{dLEC} mice. Frozen sections of the meanings were subjected to RNAscope^{TM} analyses following the manufacturer's instructions. The mRNA level of Piezo1 (green) and Prox1 (red) was detected by their specific probes. Scale bars, 20 µm. Note that the green Piezo1 signals are visible in mLVs of the control and Piezo1^{TG_LEC} mouse sections but lacking in mLVs of Piezo1^{dLEC} sections. (**b**) Graph showing the mean intensity of Piezo1 mRNA signal per Prox1-positive lymphatic vessels. n=3-5 mice/group Statistics: Kruskal-Wallis H test, p<0.0001, followed by Bonferroni correction method (statistical significance, p<0.0167). Data are presented as mean values +/- SEM.

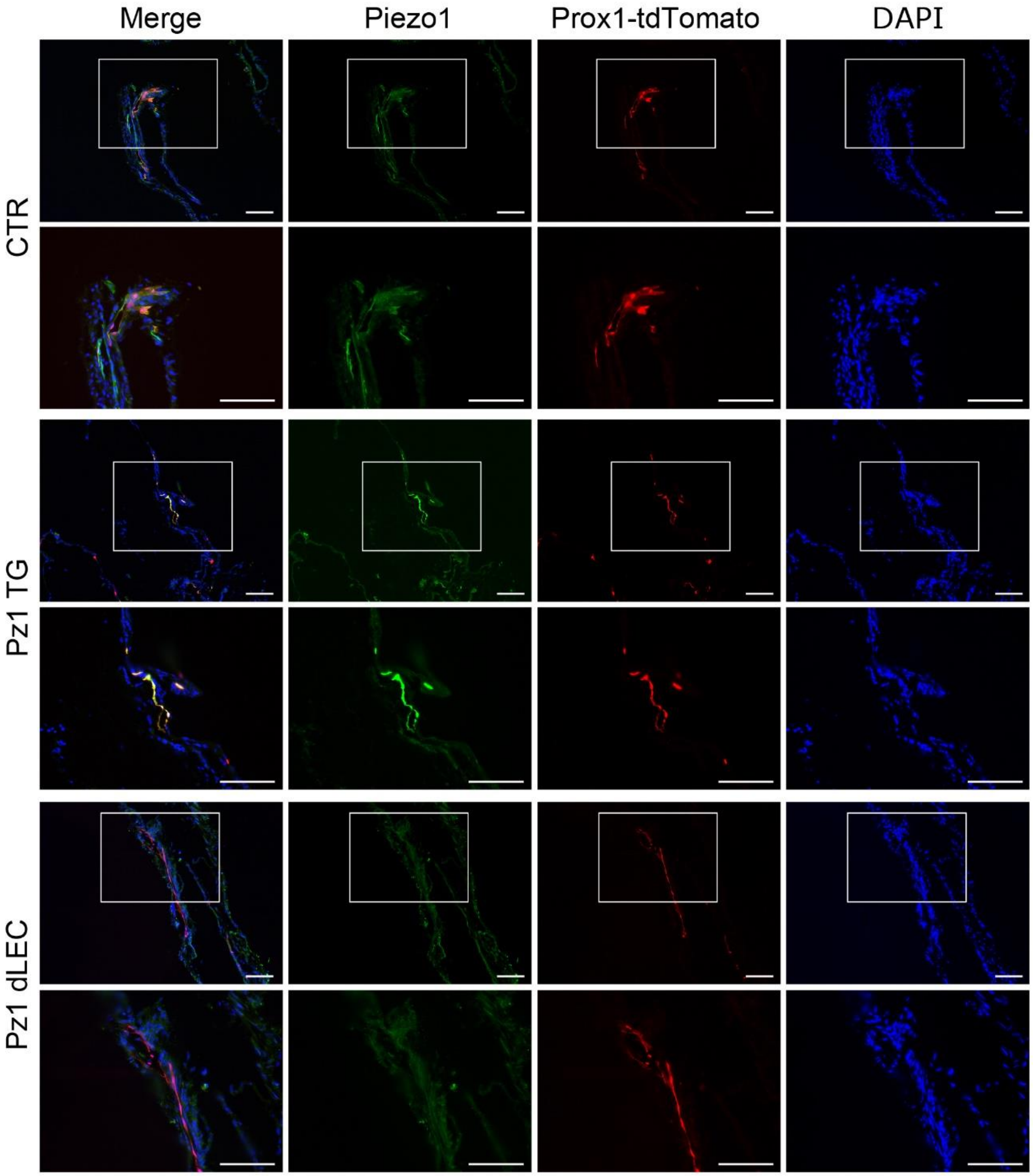

Supplementary Fig. 2 | Expression of Piezo1 Protein in Mouse Meningeal Lymphatic Vessels. *In vivo*-specificity test of the rabbit anti-Piezo1 polyclonal antibody. Frozen sections of the meninges were prepared from wild-type control (CTR), Piezo1^{TG_LEC}, and Piezo1^{dLEC} mice, all of which carry the *Prox1-tdTomato* reporter allele for precise visualization (red) of lymphatics. Sections were stained with rabbit anti-Piezo1 polyclonal antibody (green) and DAPI (blue), and all color channels were merged. Scale bars, 100 µm. Boxed areas were enlarged. Note that the green Piezo1 signals are visible in mLVs of the control and Piezo1^{TG_LEC} mouse sections but lacking in mLVs of Piezo1^{dLEC} sections. n=3-5 mice/group.

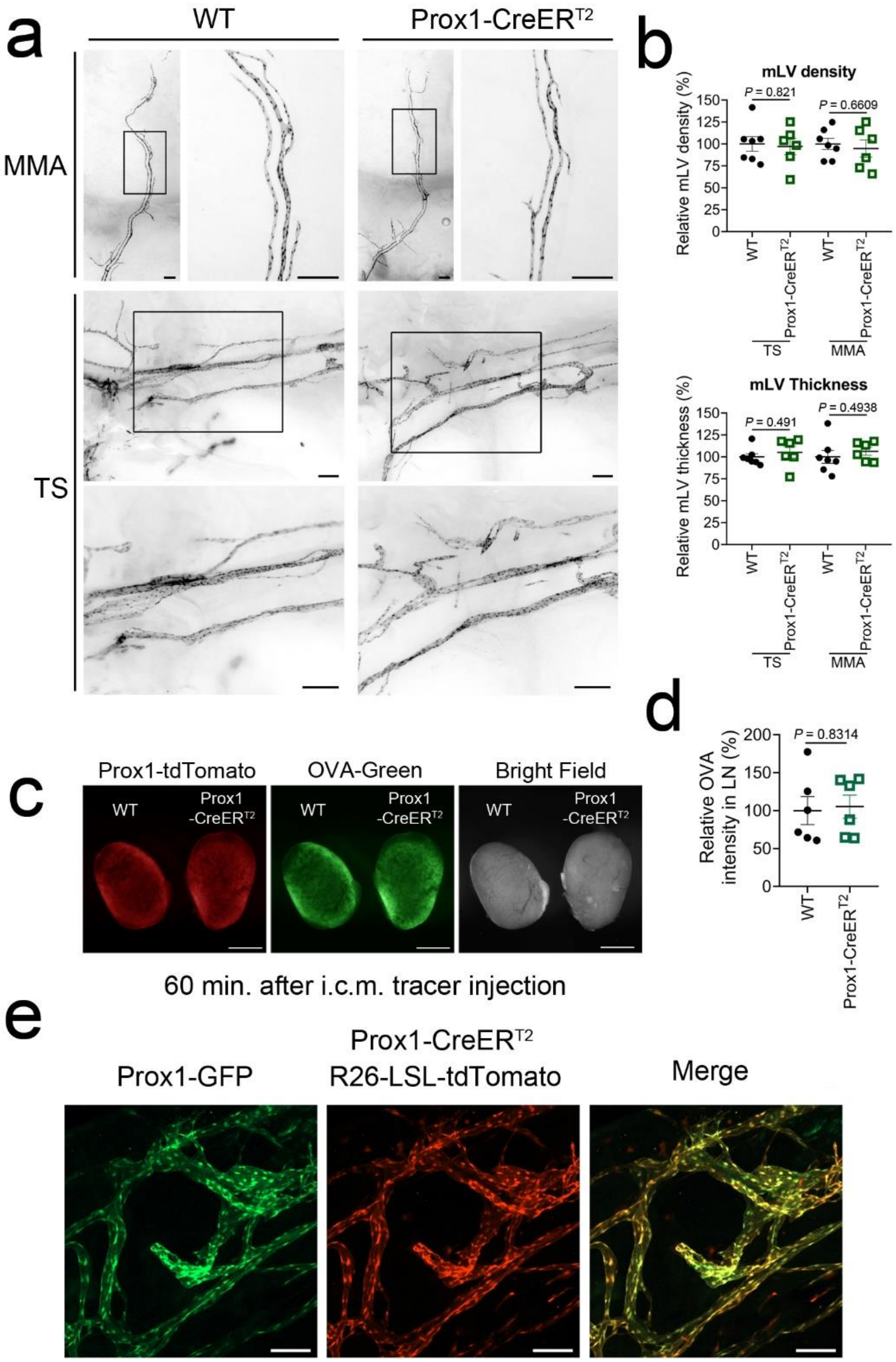

Supplementary Fig. 3 | Prox1-promoter-driven Expression of Cre Recombinase Does Not Affect Lymphatic Morphology and Drainage Function. (a-d) Adult wild-type control (Prox1-tdTomato) mice and lymphatic CreER^{T2}-expressing mice (Prox1-CreER^{T2}; Prox1-tdTomato) were i.p. injected with Tamoxifen (50 mg/kg, twice, three days apart) at the age of 7 weeks. (**a**) mLV images were captured 6 days after the first Tamoxifen injection. Scale bars: 200 µm. (**b**) Lymphatic vascular density and thickness were quantified in both control and Prox1-CreERT2 mice groups (n=6~7) for comparison. Statistics: two-tailed *t*-test. (**c**, **d**) A brain fluid tracer (OVA-Green: Ovalbumin, Alexa Fluor™ 488 Conjugate) was i.c.m. injected, and the cervical lymph nodes (LNs) were collected and subjected to imaging 60 minutes following the injection of the tracer. Scale bars: 500 µm. (**d**) The OVA intensity in LNs was quantified (n=6 mice). Each data point represents the sum of the fluorescence intensity of LNs on the right and left sides of a single mouse. n=6-7 mice/group. Statistics: two-tailed *t*-test. (**e**) Specificity test of the Cre-mediated recombination event in mLVs. The adult mice (R26-tdTomato; Prox1-CreER^{T2}; Prox1-EGFP) were i.p. injected with Tamoxifen (50 mg/kg, twice, three days apart) at the age of 10 weeks. mLV images were captured 6 days after the first Tamoxifen injection (n=5 mice). Expression of tdTomato protein mediated by Prox1-CreER^{T2} overlaps the EGFP signal expressed by Prox1-EGFP reporter. n=5 mice/group. Scale bars: 100 µm. Data are presented as mean values +/- SEM.

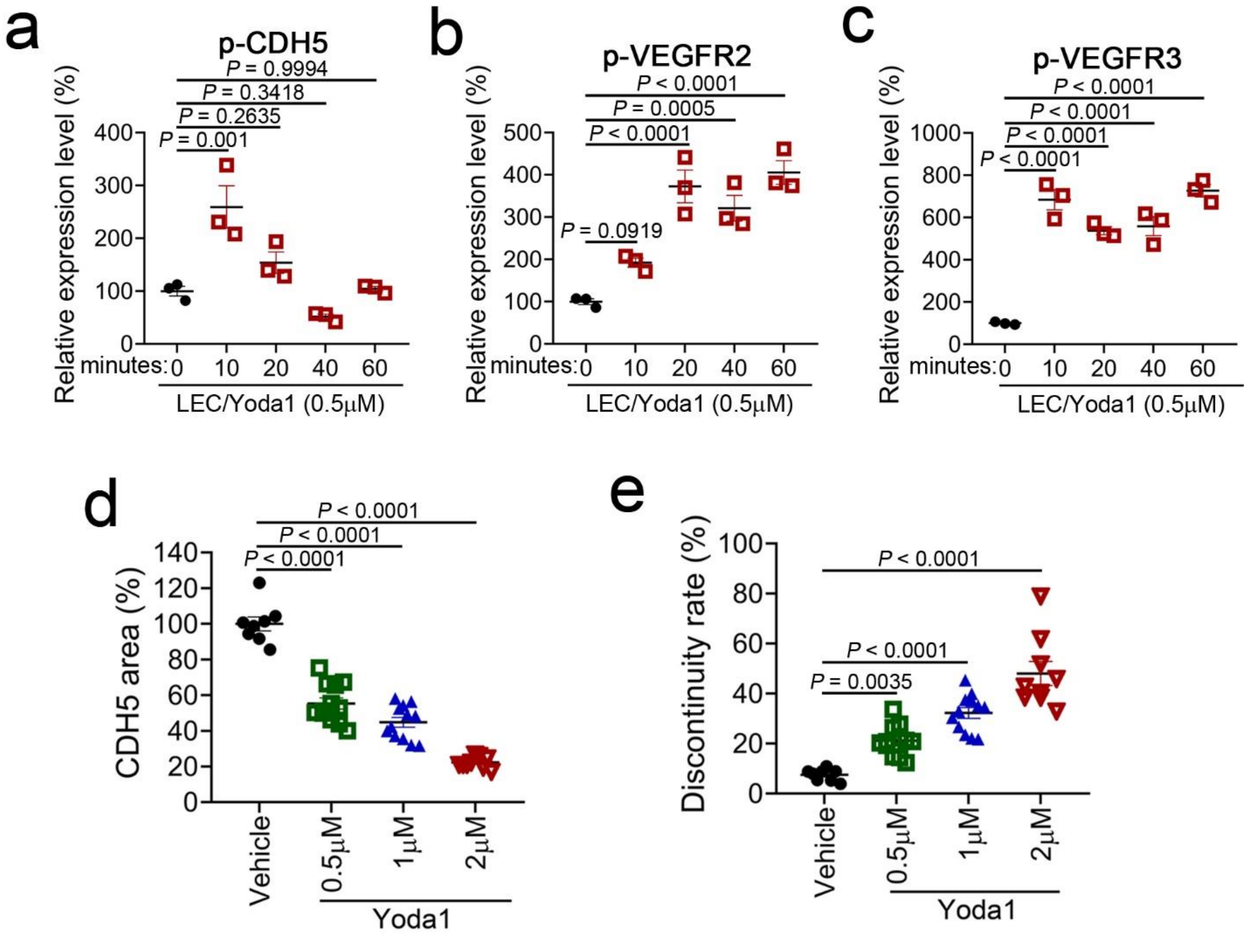

Supplementary Fig. 4 | Measurement of Western Blot Band Intensity and Cell Junction Irregularities Presented in Fig. 4. (**a-c**) The relative band intensities of phosphorylated proteins relative to the whole proteins are provided for CDH5 (**a**), VEGFR2 (**b**), and VEGFR3 (**c**) presented in **Fig. 4a** (n=3 independent samples). (**d, e**) Quantification of data shown in **Fig. 4b**: Relative CDH5-positive junctional area (**d**) and the junctional discontinuity rate (**e**) in cultured LECs treated with the vehicle or Yoda1 at the indicated concentrations. n=8-12 (n=1 means total CDH5 area or CDH5 discontinuity rate in 100 μm^2. Data are acquired from 4 independent experiments.). Statistics: one-way ANOVA, p=0.0004 (**a**); p<0.0001 (**b-e**), followed by Dunnett's multiple comparison test. Data are presented as mean values +/- SEM.

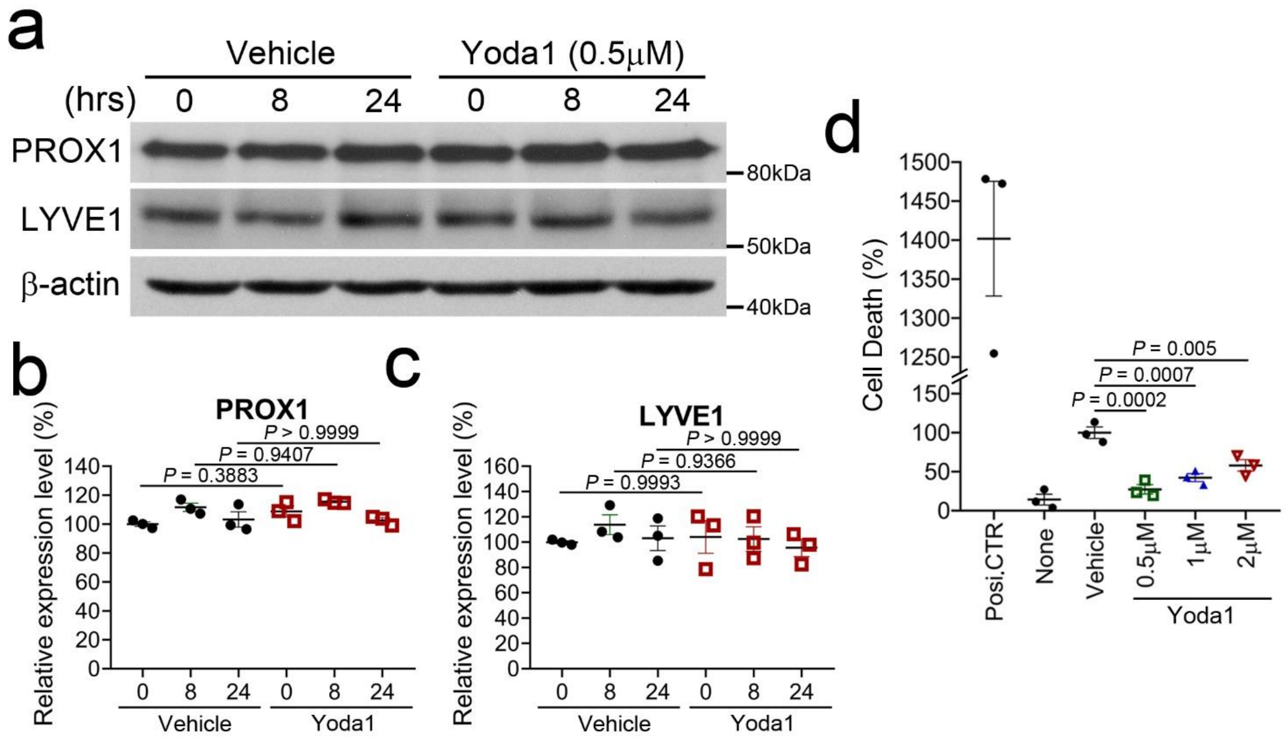

Supplementary Fig. 5 | Modulation of Prox1 and LYVE1 Expression and Cellular Toxicity by Yoda1. (**a-c**) Human primary LECs were treated with the vehicle or Yoda1 (0.5 µM) and incubated for 8 and 24 hours before harvesting the cell lysates. Western blotting was performed to detect the expression of Prox1 and LYVE1 (**a**). The relative expression level of Prox1 (**b**) and LYVE1 (**c**) was quantified. Statistics: one-way ANOVA, p=0.0267 (**b**); p=0.7899 (**c**), followed by Tukey's multiple comparison test. (**d**) Primary LECs were incubated in a low (1%) serum media with Yoda1 (0.5, 1, or 2 µM). After 8 hours, cell death was measured using a Cell Death Detection ELISA Kit (Roche) following the manufacturer's instructions. Positive control (CTR) is the cells treated with a hypertonic solution. Statistics: one-way ANOVA, p=0.0003, followed by Dunnett's multiple comparison test. Experiments were repeated three times. Data are presented as mean values +/- SEM.

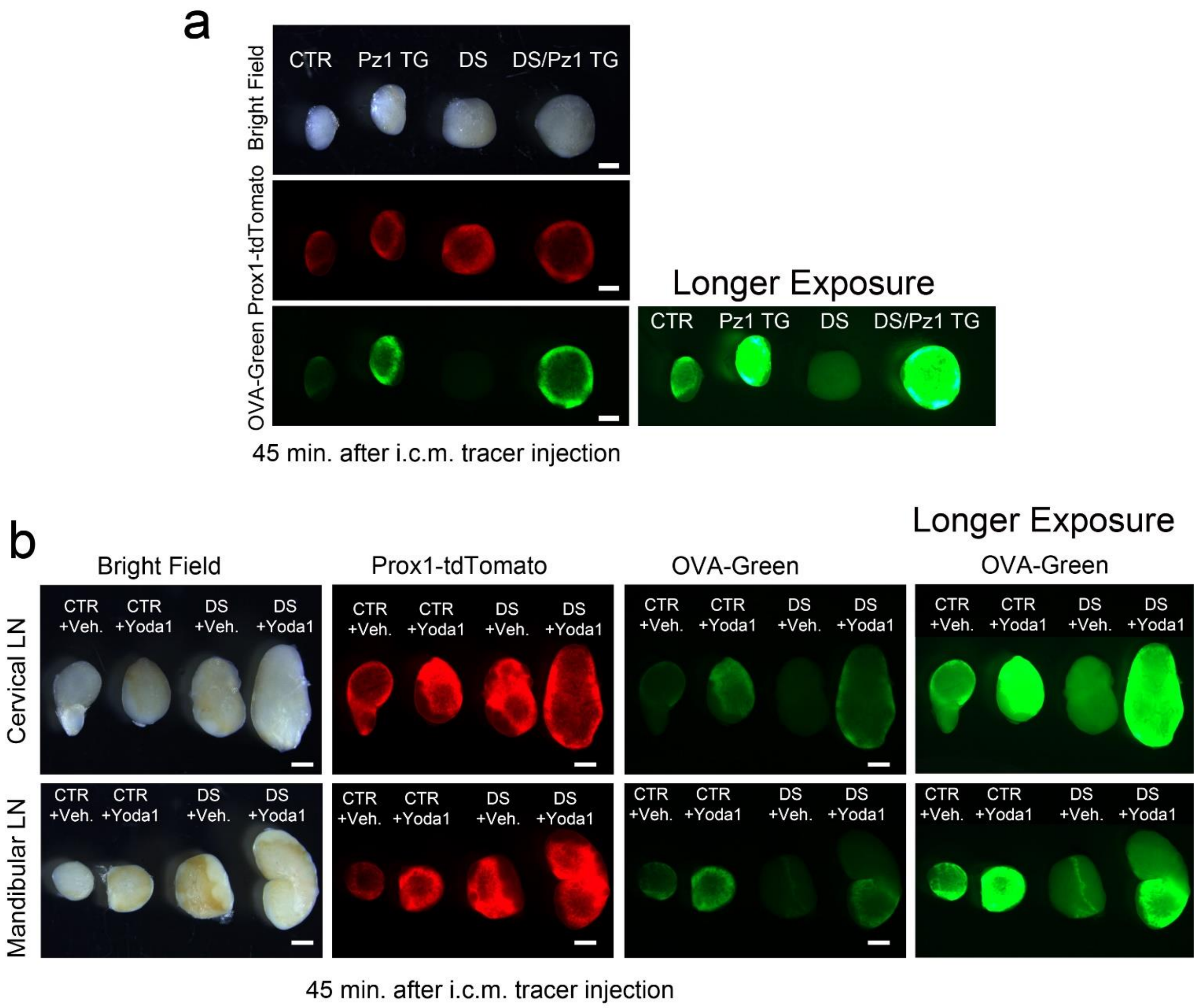

Supplementary Fig. 6 | Supplementary Exposure Images of Brain Tracer Drainage into Lymph Nodes in DS Mice. Fluorescence images taken following an Supplementary exposure period provide confirmation of the delayed drainage of brain tracer in Dp(16) DS mice, which was not apparent in the shorter-exposure images presented in **Fig. 9b** (**a**) and **Fig. 10b** (b). n=5-9 mice/group. Scale bars: 500 µm.

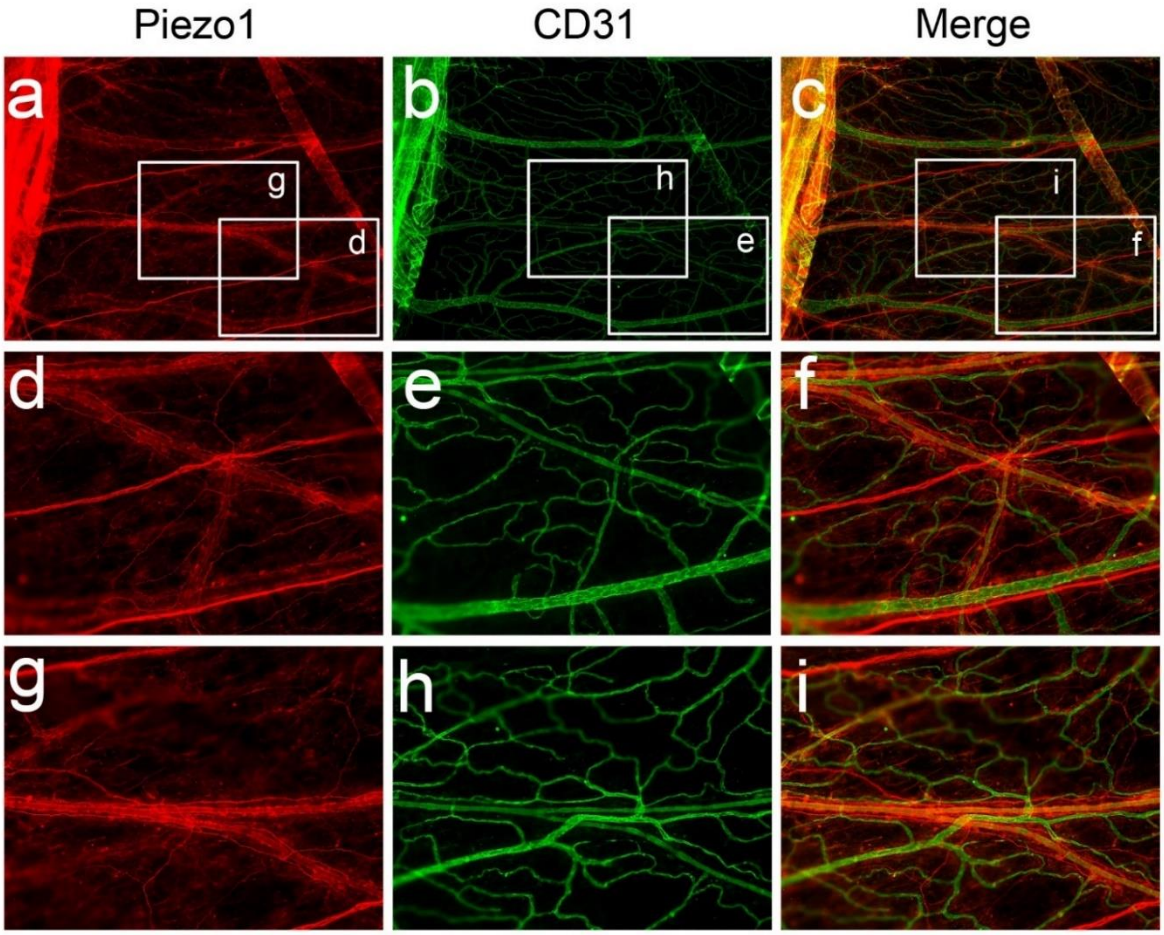

Supplementary Fig. 7 | Expression of Piezo1 in Non-Lymphatic Cells. Meninges, isolated from wild-type adult mice (6 weeks old), were whole-mount stained with anti-CD31 (green) and anti-Piezo1 (red) antibodies (**a**, **b**). The resulting color channels were merged (**c**). Boxed areas are enlarged (**d-i**). Scale bars: 200 µm. Experiments were repeated three times.

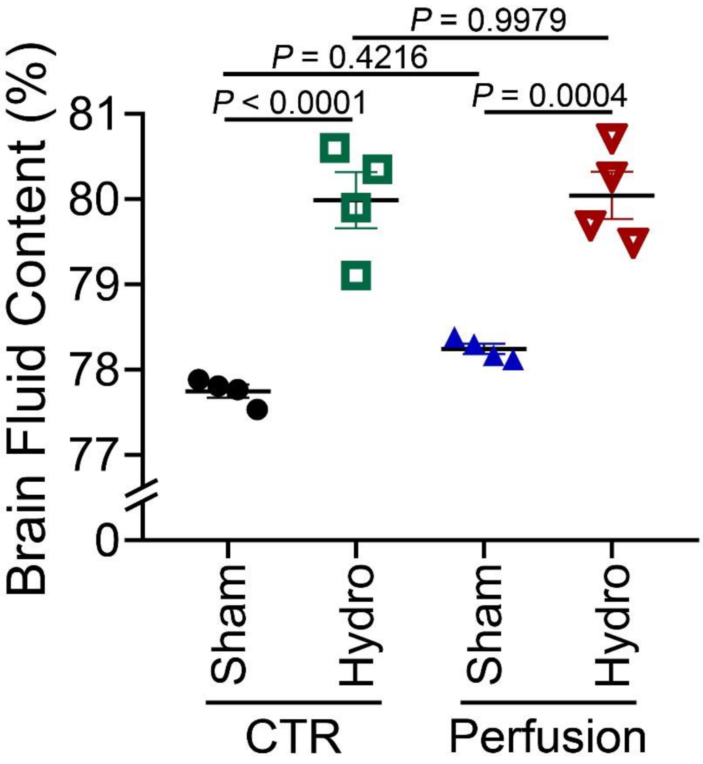

Supplementary Fig. 8 | Brain Fluid Content Was Not Affected by Transcardiac Perfusion Before Brain Harvest. The kaolin-based hydrocephalus model was induced in adult wild-type mice (7 weeks old). After five days, the mice were anesthetized, and transcardiac perfusion was performed using PBS before collecting the brains and measuring brain fluid contents. n=4 mice/group, Statistics: one-way ANOVA, p<0.0001, followed by Tukey's multiple comparison test. Data are presented as mean values +/- SEM.

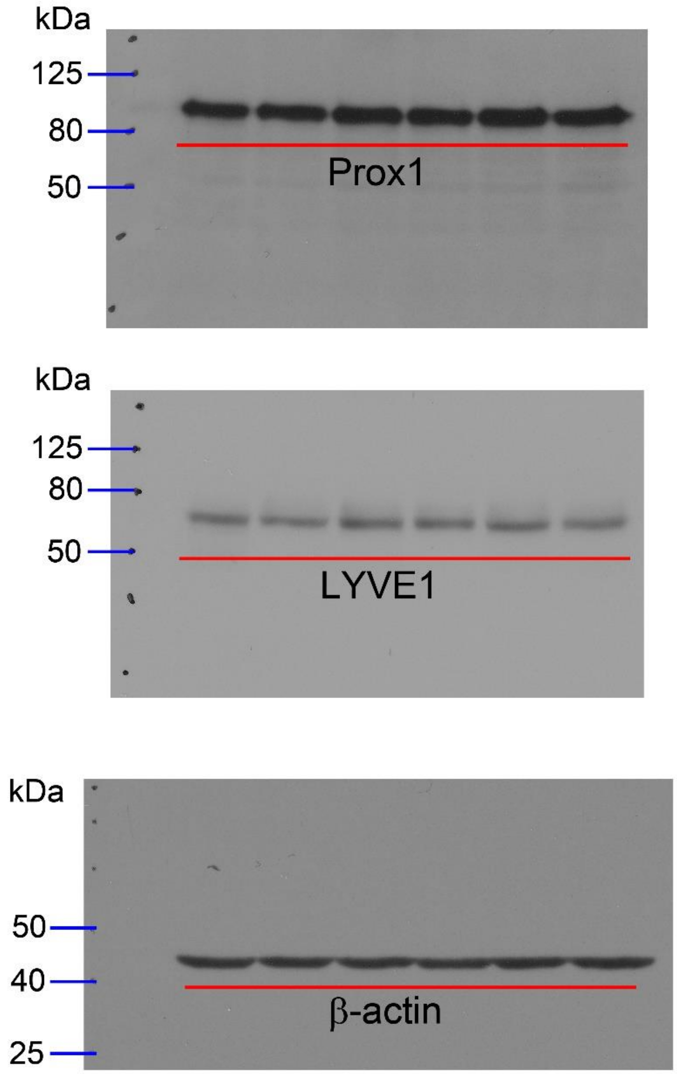

Unprocessed blots of supplementary Fig. 5a
