# Supplementary material for: Piezo1 regulates meningeal lymphatic vessel drainage and alleviates excessive CSF accumulation
Source: Nat Neurosci. 2024 Mar 25;27(5):913–26. doi: 10.1038/s41593-024-01604-8 (PMC11088999; doi:10.1038/s41593-024-01604-8)
Supplement: Supplementary file 2 — Reporting Summary [file 41593_2024_1604_MOESM2_ESM.pdf]

Corresponding author(s): Young-Kwon Hong

Last updated by author(s): Jan 30, 2024

Reporting Summary

Nature Portfolio wishes to improve the reproducibility of the work that we publish. This form provides structure for consistency and transparency in reporting. For further information on Nature Portfolio policies, see our Editorial Policies and the Editorial Policy Checklist.

Statistics

For all statistical analyses, confirm that the following items are present in the figure legend, table legend, main text, or Methods section.

n/a	Confirmed	
☐	☒	The exact sample size (*n*) for each experimental group/condition, given as a discrete number and unit of measurement
☐	☒	A statement on whether measurements were taken from distinct samples or whether the same sample was measured repeatedly
☐	☒	The statistical test(s) used AND whether they are one- or two-sided<br>*Only common tests should be described solely by name; describe more complex techniques in the Methods section.*
☒	☐	A description of all covariates tested
☐	☒	A description of any assumptions or corrections, such as tests of normality and adjustment for multiple comparisons
☐	☒	A full description of the statistical parameters including central tendency (e.g. means) or other basic estimates (e.g. regression coefficient) AND variation (e.g. standard deviation) or associated estimates of uncertainty (e.g. confidence intervals)
☐	☒	For null hypothesis testing, the test statistic (e.g. *F*, *t*, *r*) with confidence intervals, effect sizes, degrees of freedom and *P* value noted<br>*Give P values as exact values whenever suitable.*
☒	☐	For Bayesian analysis, information on the choice of priors and Markov chain Monte Carlo settings
☒	☐	For hierarchical and complex designs, identification of the appropriate level for tests and full reporting of outcomes
☐	☒	Estimates of effect sizes (e.g. Cohen's *d*, Pearson's *r*), indicating how they were calculated

Our web collection on statistics for biologists contains articles on many of the points above.

Software and code

Policy information about availability of computer code

Data collection	LabVIEW 2020 (EMERSON), Zen 2.6 (Carl Zeiss), and Leica Application Suite X 3.7.6 (Leica)
Data analysis	Tracer, meningeal lymphatic vessels, and western band density was analyzed by ImageJ 1.52a and open-access software Fiji 2.10.0 (http://fiji.sc.)<br>Brain ventricle volume was analyzed by Multi-image Analysis GUI (Mango, version 4.1).<br>Using Tracker 6.0.10, the movement of the mouse was traced, and total distance, velocity, and time spent in the center were quantified.<br>GraphPad Prism 8 and SPSS 12.0 (IBM) were used for statistical analysis.

For manuscripts utilizing custom algorithms or software that are central to the research but not yet described in published literature, software must be made available to editors and reviewers. We strongly encourage code deposition in a community repository (e.g. GitHub). See the Nature Portfolio guidelines for submitting code & software for further information.

Data

Policy information about availability of data

All manuscripts must include a data availability statement. This statement should provide the following information, where applicable:

- Accession codes, unique identifiers, or web links for publicly available datasets
- A description of any restrictions on data availability
- For clinical datasets or third party data, please ensure that the statement adheres to our policy

All data supporting the findings of this study are available within the paper and supplementary information.

Research involving human participants, their data, or biological material

Policy information about studies with human participants or human data. See also policy information about sex, gender (identity/presentation), and sexual orientation and race, ethnicity and racism.

Reporting on sex and gender	N/A
Reporting on race, ethnicity, or other socially relevant groupings	N/A
Population characteristics	N/A
Recruitment	N/A
Ethics oversight	N/A

Note that full information on the approval of the study protocol must also be provided in the manuscript.

Field-specific reporting

Please select the one below that is the best fit for your research. If you are not sure, read the appropriate sections before making your selection.

☒ Life sciences ☐ Behavioural & social sciences ☐ Ecological, evolutionary & environmental sciences

For a reference copy of the document with all sections, see nature.com/documents/nr-reporting-summary-flat.pdf

Life sciences study design

All studies must disclose on these points even when the disclosure is negative.

Sample size	Sample size was predicted and chosen by the power analysis using G*Power (hhu). The parameters for the analysis were Effect size (Cohen's d or Cohen's f) = 0.8-2, α err prob (Significance level) = 0.05, Power (1-β err prob) = 0.8, and Allocation ratio N2/N1 = 1. Effect sizes were calculated based on pilot experiments.
Data exclusions	No data were excluded.
Replication	All experiments were replicated based on the calculated sample size and indicated in figures and manuscript.
Randomization	Animals were randomly assigned to each experimental group.
Blinding	Data collection was not performed blind to the conditions of the experiment, but data analysis was blindly performed by third person following surgery or sample collection.

Reporting for specific materials, systems and methods

We require information from authors about some types of materials, experimental systems and methods used in many studies. Here, indicate whether each material, system or method listed is relevant to your study. If you are not sure if a list item applies to your research, read the appropriate section before selecting a response.

Materials & experimental systems

n/a	Involved in the study	
☐	☒	Antibodies
☐	☒	Eukaryotic cell lines
☒	☐	Palaeontology and archaeology
☐	☒	Animals and other organisms
☒	☐	Clinical data
☒	☐	Dual use research of concern
☒	☐	Plants

Methods

n/a	Involved in the study	
☒	☐	ChIP-seq
☒	☐	Flow cytometry
☐	☒	MRI-based neuroimaging

Antibodies

Antibodies used

For Western blotting, we used α-β-actin (Sigma-Aldrich, A5441), α-p-CDH5 (Try658) (ThermoFisher Scientific, 44-1144G), α-p-CDH5 (Try685) (ECM Biosciences, CP1981), α-CDH5 (Santa Cruz Biotechnology, sc-6458), α-p-AKT(Ser473) (Cell Signaling Technology, #4060), α-AKT (Cell Signaling Technology, #9272), α-eNOS (Cell Signaling Technology, #32027), α-p-eNOS (Ser1177) (Cell Signaling Technology, # 9571), α-VEGFR2 (R&D Systems, AF357), α-p-VEGFR2 (Tyr1054/1059) (Invitrogen, 44-1047G), α-VEGFR3 (Santa Cruz Biotechnology, sc-321), α-p-VEGFR3 (Tyr1230/1231) (Cell Applications, Inc., CY1115), α-Prox1 (DSHB, AB2619013), and α-LYVE1 (R&D systems, AF2089) antibodies. Anti-Piezo1 rabbit polyclonal antibody was generated by the authors (GenScript, Inc.).
For immunofluorescence staining, anti-LYVE1 (Angiobio, 11-034), anti-Pdpn (DSHB, AB531893), anti-VEGFR3 (R&D systems, AF743). we used a rabbit anti-Piezo1 antibody generated by the authors (Genscript Inc.). Alexa-fluorescence secondary antibodies (Invitrogen, A21206, A21113, A21207, A11058) were used for immunofluorescence staining.

Validation

All commercial antibodies are prevalidated for the species (human and mouse) and applications (immunostaing and Western blotting) by the supplier. The quality control informations are stated on the website of the manufacture as follows.
α-β-Actin validated by Western blotting for human and mouse: https://www.sigmaaldrich.com/US/en/substance/monoclonalantibetaactinantibodyproducedinmouse1234598765
α-p-CDH5 (Try658) validated by Western blotting for human: https://www.thermofisher.com/antibody/product/Phospho-VE-cadherin-Tyr658-Antibody-Polyclonal/44-1144G
α-p-CDH5 (Try685) validated by Western blotting for human: https://ecmbio.com/products/cp1981
α-CDH5 validated by Western blotting for human: https://www.scbt.com/p/ve-cadherin-antibody-c-19
α-p-AKT(Ser473) validated by Western blotting for human: https://www.cellsignal.com/products/primary-antibodies/phospho-akt-ser473-d9e-xp-rabbit-mab/4060
α-AKT validated by Western blotting for human: https://www.cellsignal.com/products/primary-antibodies/akt-antibody/9272
α-eNOS validated by Western blotting for human: https://www.cellsignal.com/products/primary-antibodies/enos-d9a5l-rabbit-mab/32027
α-p-eNOS (Ser1177) validated by Western blotting for human: https://www.cellsignal.com/products/primary-antibodies/phospho-enos-ser1177-antibody/9571
α-VEGFR2 validated by Western blotting for human: https://www.rndsystems.com/products/human-vegfr2-kdr-flk-1-antibody_af357
α-p-VEGFR2 (Tyr1054/1059) validated by Western blotting for human: https://www.thermofisher.com/antibody/product/Phospho-VEGF-Receptor-2-Tyr1054-Tyr1059-Antibody-Polyclonal/44-1047G
α-VEGFR3 validated by Western blotting for human: https://www.scbt.com/p/flt-4-antibody-c-20
α-p-VEGFR3 (Tyr1230/1231) validated by Western blotting for human: https://www.cellapplications.com/
α-Prox1 validated by Western blotting for human: https://dshb.biology.uiowa.edu/PCRP-PROX1-1A6
α-LYVE1 (R&D systems, AF2089) validated by Western blotting for human: https://www.rndsystems.com/products/human-lyve-1-antibody_af2089
α-LYVE1 (Angiobio, 11-034) validated by immunofluorescence staining for mouse: https://www.angiobio.com/new/product.php?pid=8
α-Pdpn validated by immunofluorescence staining for mouse: https://dshb.biology.uiowa.edu/8-1-1
α-VEGFR3 validated by immunofluorescence staining for mouse: https://www.rndsystems.com/products/mouse-vegfr3-flt-4-antibody_af743
Piezo1 antibody was validated by in vitro knock down and overexpression test and by immunofluorescence staining of Piezo1 fl/fl and Piezo1 tg mouse section.
Alexa-fluorescence secondary antibodies (Invitrogen, A21206, A21113, A21207, A11058):
A21206 validated by immunofluorescence staining for Rabbit IgG: https://www.thermofisher.com/antibody/product/Donkey-anti-Rabbit-IgG-H-L-Highly-Cross-Adsorbed-Secondary-Antibody-Polyclonal/A-21206
A21113 validated by immunofluorescence staining for Syrian Hamster IgG: https://www.thermofisher.com/antibody/product/Goat-anti-Syrian-Hamster-IgG-H-L-Cross-Adsorbed-Secondary-Antibody-Polyclonal/A-21113
A21207 validated by immunofluorescence staining for Rabbit IgG: https://www.thermofisher.com/antibody/product/Donkey-anti-Rabbit-IgG-H-L-Highly-Cross-Adsorbed-Secondary-Antibody-Polyclonal/A-21207
A11058 validated by immunofluorescence staining for Goat IgG: https://www.thermofisher.com/antibody/product/Donkey-anti-Goat-IgG-H-L-Cross-Adsorbed-Secondary-Antibody-Polyclonal/A-11058

Eukaryotic cell lines

Policy information about cell lines and Sex and Gender in Research

Cell line source(s)

Human dermal lymphatic endothelial cells (LECs) were isolated from identified human foreskins (male). HEK293 cell line was purchased from ATCC.

Authentication	The isolated human dermal lymphatic endothelial cells were stained and authenticated with antibodies against lymphatic endothelial marker genes such as Prox1, PDPN, and VEGFR3. The purchased primary cells and cell line were prevalidated by the supplier.
Mycoplasma contamination	LECs and HEK293 cells were regularly tested by PCR assay (ATCC, 30-1012K) and confirmed that the cells were negative for mycoplasma contamination.
Commonly misidentified lines (See ICLAC register)	No commonly misidentified cell lines were used in the study

Animals and other research organisms

Policy information about studies involving animals; ARRIVE guidelines recommended for reporting animal research, and Sex and Gender in Research

Laboratory animals	C57BL/6J (The Jackson Laboratory, Bar Harbor, ME), Prox1-EGFP and Prox1-tdTomato (Mutant Mouse Resource and Research Centers, Davis, California, USA), ROSA26-LSL-tdTomato (The Jackson Laboratory), Prox1-CreERT2 (a gift from Dr. Taija Mäkinen, Uppsala University, Uppsala, Sweden), Piezo1flox/flox (Piezo1tm2.1Apat/J, The Jackson Laboratory), Piezo1 transgenic line (CAG-LSL-Piezo1) (previously generated and reported by the authors), Dp(16)1Yey/+ (B6.129S7-Dp(16Lipi-Zbtb21)1Yey/J, The Jackson Laboratory, USA). Mice were aged 2 days to 10 months old.
Wild animals	No wild animals were used in the study.
Reporting on sex	Both male and female mice were used in this study. The sex and number of mice used in each experiment were shown in Extended Data Table 1.
Field-collected samples	No field collected samples were used in the study.
Ethics oversight	Mouse experiments were performed following the University of Southern California Institutional Animal Care and Use Committee (IACUC).

Note that full information on the approval of the study protocol must also be provided in the manuscript.

Magnetic resonance imaging

Experimental design

Design type	Structural MRI
Design specifications	In this study, mouse brain was scanned under anesthesia to measure brain ventricle volume. No tasks or trials was given to the subjects during the MRI scan.
Behavioral performance measures	Behavioral performance measures was not required for this study because this study purposed to measure brain ventricle volume of mice under anesthesia. No tasks or trials was given to the subjects during the MRI scan.

Acquisition

Imaging type(s)	Structural
Field strength	7 Telsa
Sequence & imaging parameters	Two-dimensional Fast Spin Echo (FSE) T2-weighted MR images were acquired in coronal and transverse orientations to identify anatomy. The transverse FSE T2-weighted imaging parameters were TR = 4500 ms, TE = 45 ms, number of averages (NA) = 3, echo train length = 7, slice thickness = 0.40 mm, field of view (FOV) = 16 mm x 16 mm, matrix size (MS) = 256 x 238, in-plane-resolution = 0.0625 x 0.0672 mm2 / pixel, number of slices = 32. The coronal FSE T2-weighted images had the following parameters: TR = 4500 ms, TE = 45 ms, number of averages (NA) = 3, echo train length = 7, slice thickness = 0.40 mm, field of view (FOV) = 18 mm x 18 mm, matrix size (MS) = 238 x 256, in-plane-resolution = 0.0756 x 0.0703 mm2 / pixel, number of slices = 32.
Area of acquisition	Whole brain

Diffusion MRI ☐ Used ☒ Not used

Preprocessing

Preprocessing software	Multi-image Analysis GUI (Mango 4.1) and FMRIB Software Library, Release 4.0
Normalization	Using Mango 4.1 plugin (Register brain) based on FLIRT (FMRIB's Linear Image Registration Tool)
Normalization template	A representative MRI scan images of each age group was used as the template for transformation.

Noise and artifact removal	Noise and artifact removal were not performed because movement of mice was minimized under anesthesia and a 20-mm diameter quadrature birdcage coil optimized for small animals was used to improve signal-to-noise ratio (SNR). In fact, using ANTs (Advanced Normalization Tools) to remove noise and artifact did not improve the quality of the original MRI data.
Volume censoring	Brain extraction tool (BET 1.1) was used to separate brain from non-brain tissues.

Statistical modeling & inference

Model type and settings	Simple univariate (2-tailed Student's t-test or one-way ANOVA).
Effect(s) tested	Brain ventricle volume was compared between control group and experimental group. The statistical significance was determined by 2-tailed t-test or one-way ANOVA.

Specify type of analysis: ☒ Whole brain ☐ ROI-based ☐ Both

Statistic type for inference<br>(See Eklund et al. 2016)	Voxel-wise
Correction	Experiment-wide multiple corrections were not applied.

Models & analysis

n/a	Involved in the study
☒	☐ Functional and/or effective connectivity
☒	☐ Graph analysis
☒	☐ Multivariate modeling or predictive analysis
