# Supplementary figures and images for: Piezo1 regulates meningeal lymphatic vessel drainage and alleviates excessive CSF accumulation
Source: Nat Neurosci. 2024 Mar 25;27(5):913–26. doi: 10.1038/s41593-024-01604-8 (PMC11088999; doi:10.1038/s41593-024-01604-8)

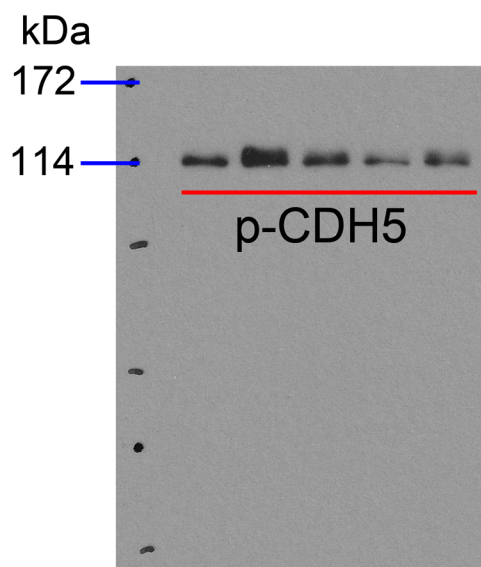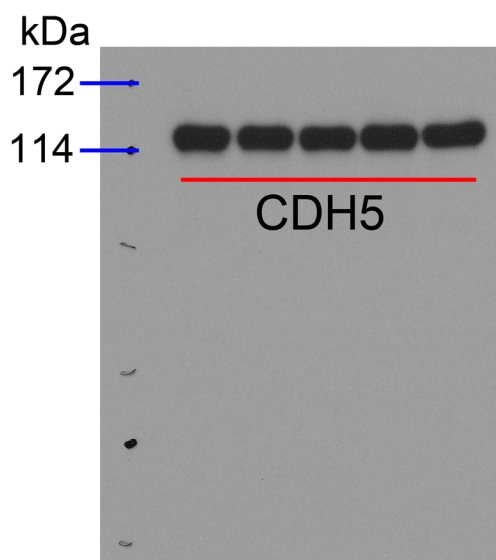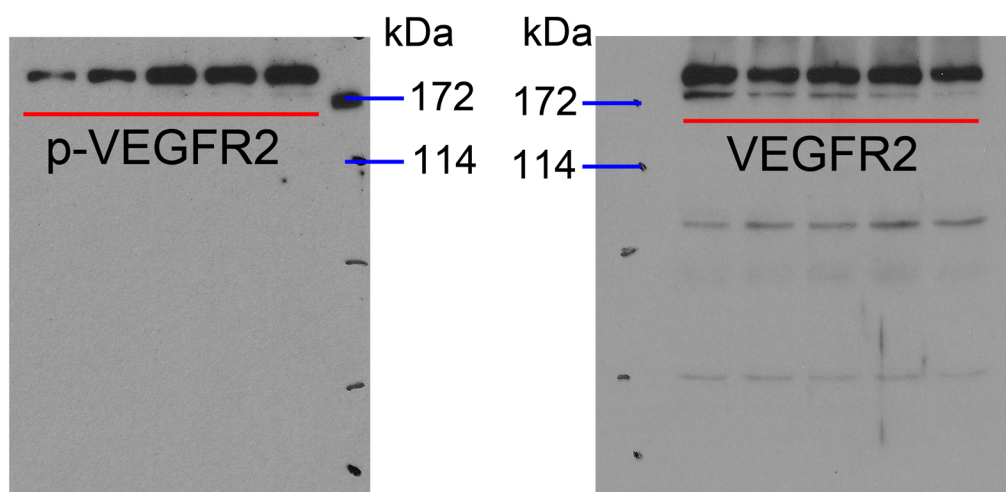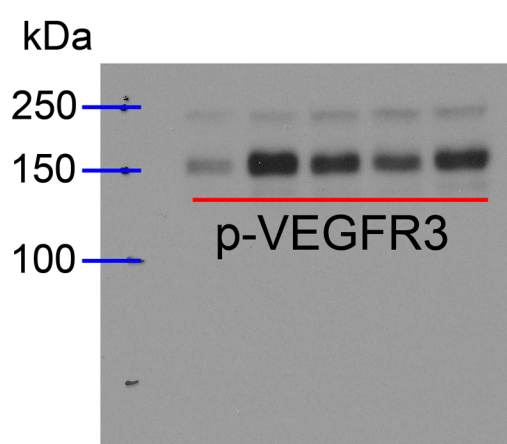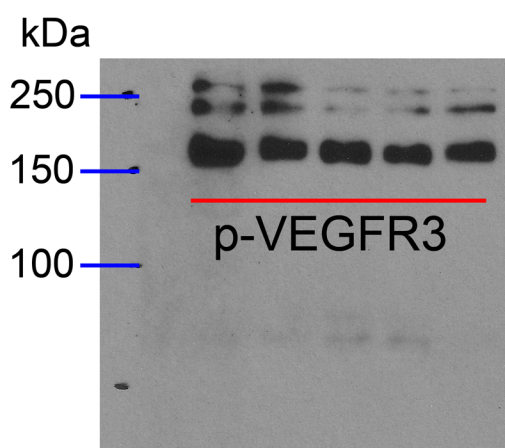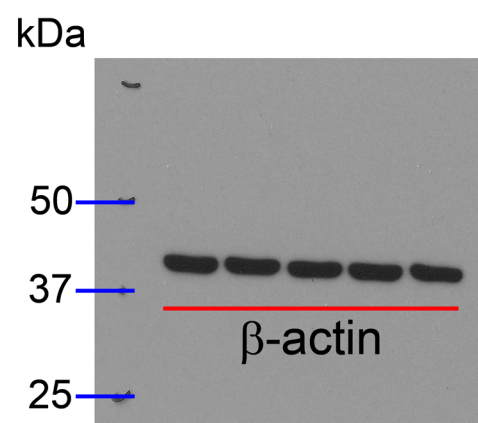

Fig. 4a

Supplement: Supplementary file 15 — Unprocessed blots. [file 41593_2024_1604_MOESM15_ESM.pdf]

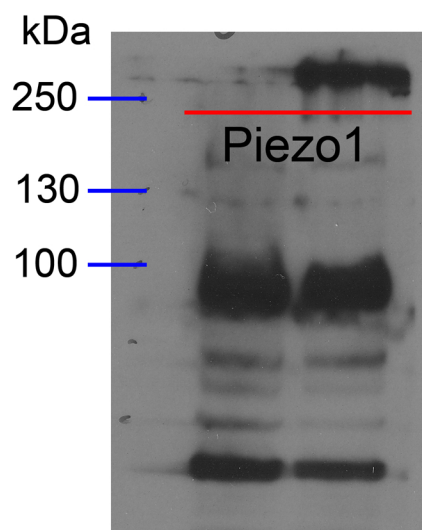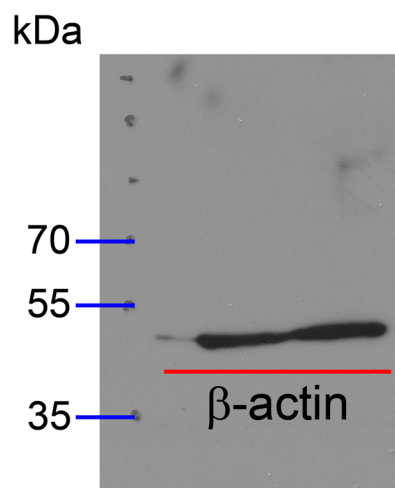

Extended Data fig. 1f

Supplement: Supplementary file 21 — Unprocessed blots. [file 41593_2024_1604_MOESM21_ESM.pdf]

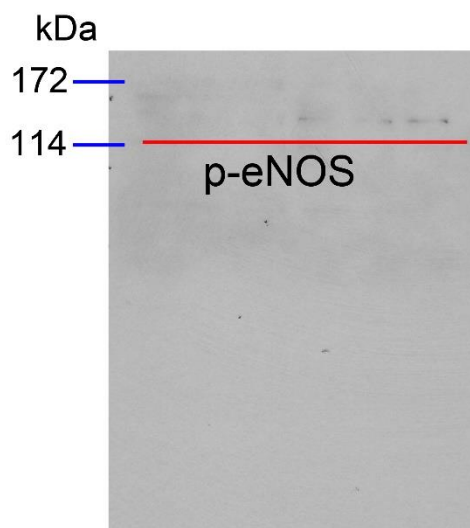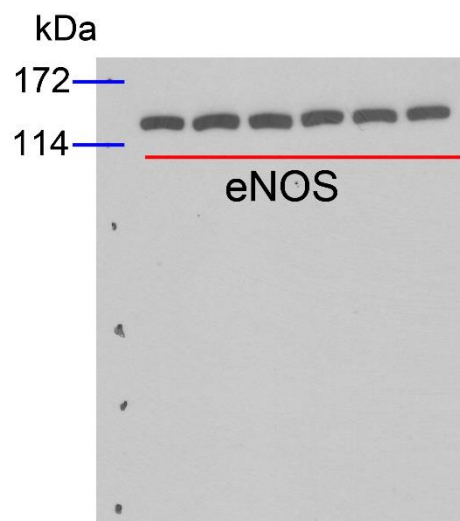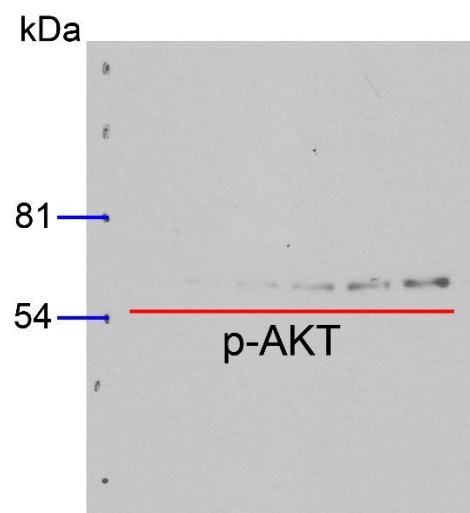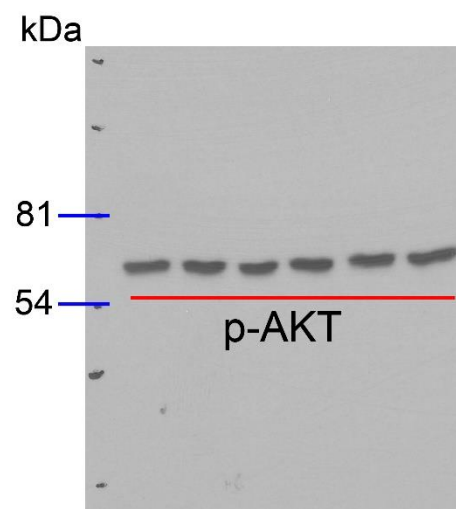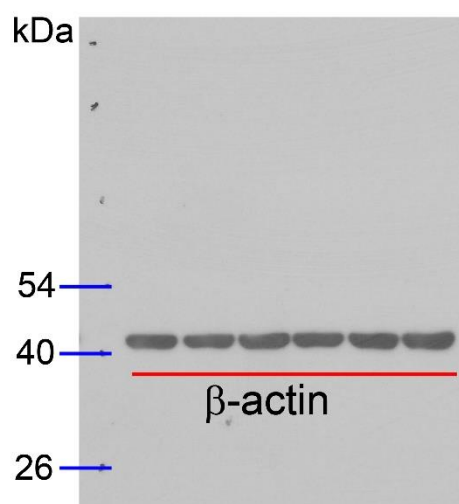

Extended Data fig. 8a

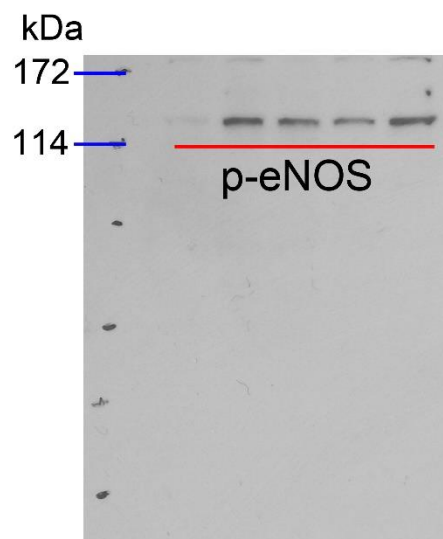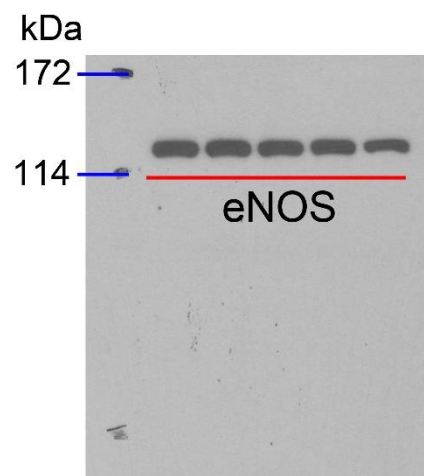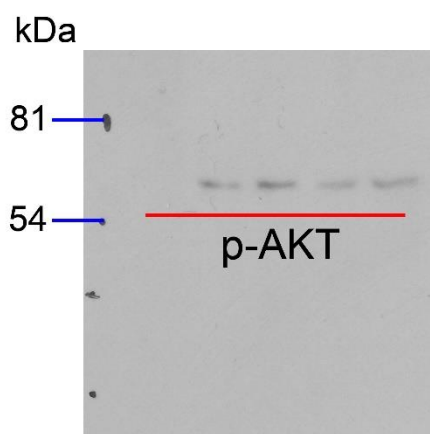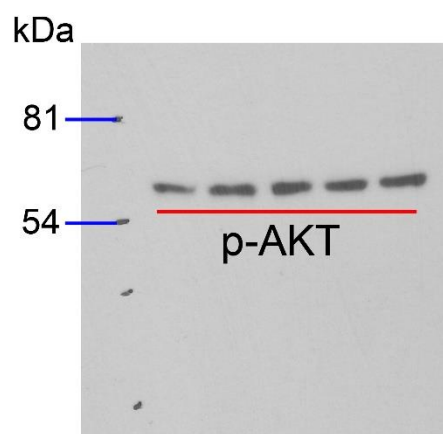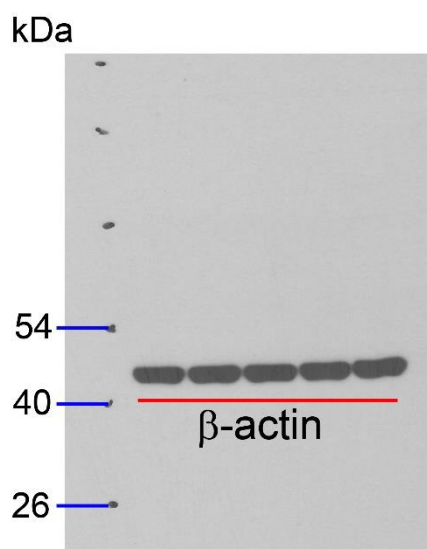

Extended Data fig. 8d

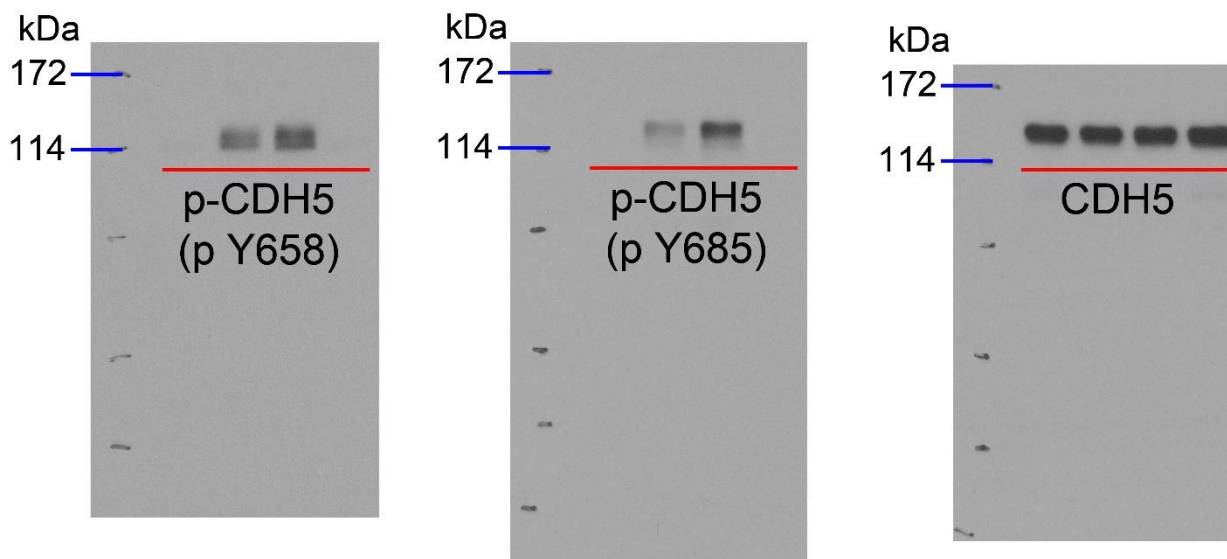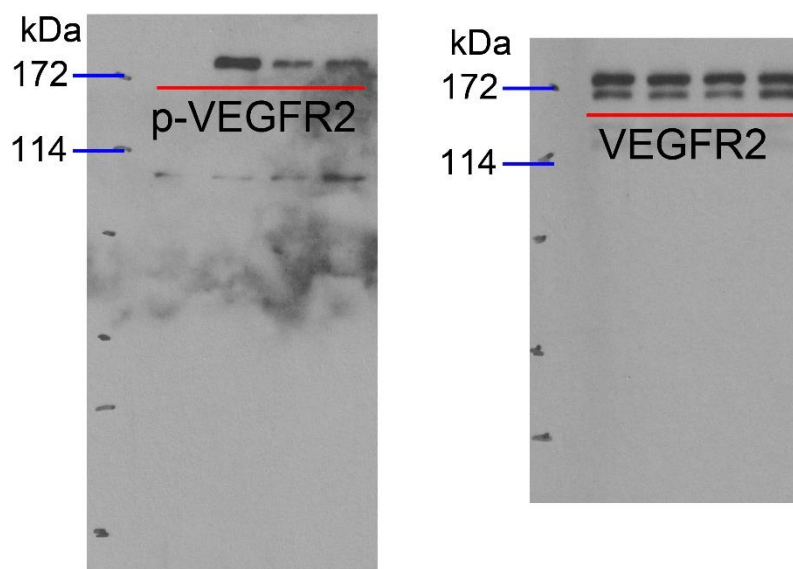

Extended Data fig. 8g

Supplement: Supplementary file 27 — Unprocessed blots. [file 41593_2024_1604_MOESM27_ESM.pdf]

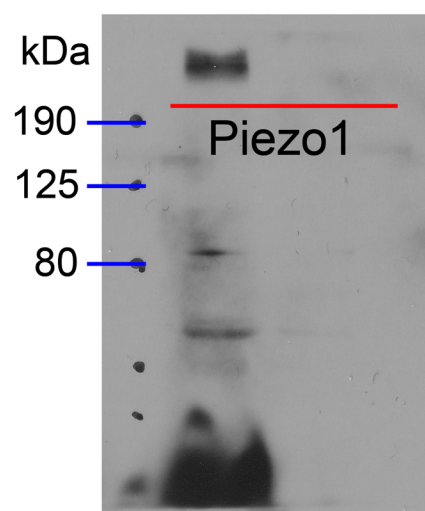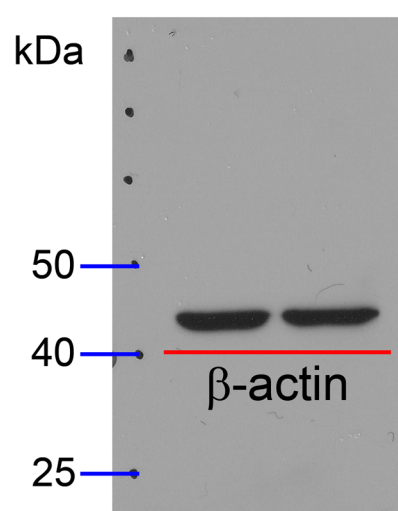

Extended Data fig. 9d

Supplement: Supplementary file 29 — Unprocessed blots. [file 41593_2024_1604_MOESM29_ESM.pdf]
